# GABRD promotes hepatocellular carcinoma progression via the IL-10RA/JAK2-STAT3 signaling axis

**DOI:** 10.1038/s41419-026-08969-7

**Published:** 2026-06-08

**Authors:** Chengcheng Huang, Weiwei Liu, Zheng Wang, Yingling Liu, Zhenfei Bi, Bin Han, Yue Yan, Shuqi Zhang, Boyu Zhu, Ziyi Zhou, Chaoqun Wang, Lu Zheng, Jing Li

**Affiliations:** 1 Key Laboratory for Targeted Therapy of Hepatopancreatobiliary Diseases of Chongqing, Department of Hepatopancreatobiliary Surgery, The Second Affiliated Hospital of Army Medical University, Chongqing, 400037 China; 2 Department of General Surgery, Xinhua Hospital, Affiliated to Shanghai Jiao Tong University School of Medicine, Shanghai, 200092 China

**Keywords:** Cancer genomics, Epithelial-mesenchymal transition, Prognostic markers

## Abstract

Hepatocellular carcinoma (HCC) is a highly aggressive malignancy with a poor prognosis, primarily owing to its capacity of metastasis, underscoring the urgent need to identify novel drivers of HCC progression. While neurotransmitter receptors are increasingly recognized as regulators of tumor biology, the role of the gamma-aminobutyric acid type A receptor delta subunit (GABRD) in HCC remains unexplored. Here, we show that GABRD is significantly upregulated in HCC tissues and cell lines, and high expression of GABRD correlates with poor prognosis in HCC patients. Loss- and gain-of-function studies demonstrated that GABRD promotes HCC cell proliferation, invasion, and migration in vitro, and accelerates tumor growth in both subcutaneous xenograft and spontaneous HCC models in vivo. Mechanistically, we find that GABRD physically interacts with the interleukin-10 receptor alpha subunit (IL-10RA). This interaction orchestrates the activation of the JAK2-STAT3 signaling axis, driving epithelial-mesenchymal transition (EMT). Inhibition of JAK2-STAT3 and IL-10RA partially reverses the tumorigenic effects of GABRD. Our study establishes the GABRD-IL-10RA-JAK2-STAT3 axis as a critical oncogenic driver in HCC and identifies GABRD as a promising therapeutic target for improving HCC outcomes.

**Figure:**
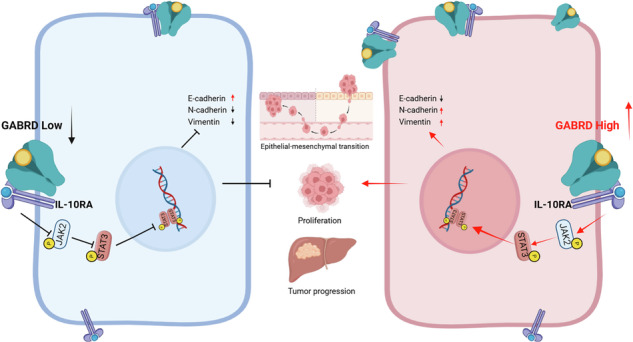

## Introduction

Hepatocellular carcinoma (HCC) is the third leading cause of cancer-related death worldwide, with over 900,000 new cases diagnosed annually [1]. Despite significant improvements in survival for some patients through surgical resection, liver transplantation, and targeted therapies, the 5-year survival rate for HCC remains at only 18%, primarily due to the high propensity of the disease to metastasize and associated chemoresistance [2, 3]. Consequently, a critical and urgent need exists to elucidate the key drivers of HCC progression and to develop clinically valuable prognostic biomarkers and therapeutic targets.

Recent studies have highlighted the critical role of neurotransmitters in tumor progression. Within the tumor microenvironment (TME), various cell types—including tumor cells, immune cells, and other stromal cells—can secrete neurotransmitters that modulate key tumor biological processes such as tumorigenesis, metastasis, and immune evasion. Moreover, signaling between neurotransmitters and their receptors is pivotal to tumor initiation and progression, representing a burgeoning area of oncological research [4–6]. For instance, norepinephrine-mediated activation of β-adrenergic receptors (β-AR) has been shown to suppress the effector functions of CD8^+^ T cells, thereby compromising the efficacy of immune checkpoint inhibitors [7]; In murine colorectal carcinoma models, elevated acetylcholine levels correlated with increased tumor proliferation and metastasis; these effects were effectively abrogated by M3 muscarinic receptor antagonists [8]; Similarly, γ-aminobutyric acid (GABA) was demonstrated to promote tumor angiogenesis and progression in lung adenocarcinoma by upregulating FGF2 expression in macrophages via GABA-A receptor signaling [9]. Collectively, these findings establish neurotransmitter receptors as central regulators of tumor biology and highlight their potential as novel therapeutic targets for improving HCC prognosis.

The GABA receptor family comprises two major classes: ligand-gated ion channels (GABA_A_ and GABA_C_) and G protein-coupled receptors (GABA_B_). GABA_A_ receptors function as heteropentameric chloride channels that mediate rapid synaptic inhibition [10]. Among the subunits of these receptors, the delta subunit (GABRD) exhibits distinctive tissue distribution and pharmacological properties. Predominantly localized to extrasynaptic regions, GABRD assembles with α6 or α4 subunits to form high-affinity receptor complexes that generate tonic inhibition, thereby fine-tuning cellular excitability [11, 12]. While dysregulation of GABRD is strongly associated with neuropsychiatric disorders such as depression and epilepsy [13, 14], emerging evidence also points to its significant involvement in oncogenesis across various cancer types. GABRD knockdown was shown to inhibit the proliferation of prostate cancer cells [15]; In esophageal squamous cell carcinoma, GABRD forms a complex with DEPDC1B to activate the PI3K/Akt/mTOR pathway, driving tumor progression [16]; In gastric cancer, GABRD accelerates tumor growth through modulation of CCND1 signaling [17]; GABRD also promotes breast cancer metastasis by activating Ca^2+^ -dependent PKC-CREB signaling [18], and meanwhile is associated with poor clinical outcomes in colorectal cancer [19–21]. Whereas, the molecular mechanisms by which GABRD regulates HCC tumorigenesis remain entirely unexplored.

In this study, we elucidate the critical role of GABRD in HCC metastasis and progression, delineate the underlying pro-tumorigenic mechanism, and present GABRD as a promising novel therapeutic target for HCC.

## Results

### GABRD is upregulated in HCC and correlates with poor prognosis

**Fig. 1:**
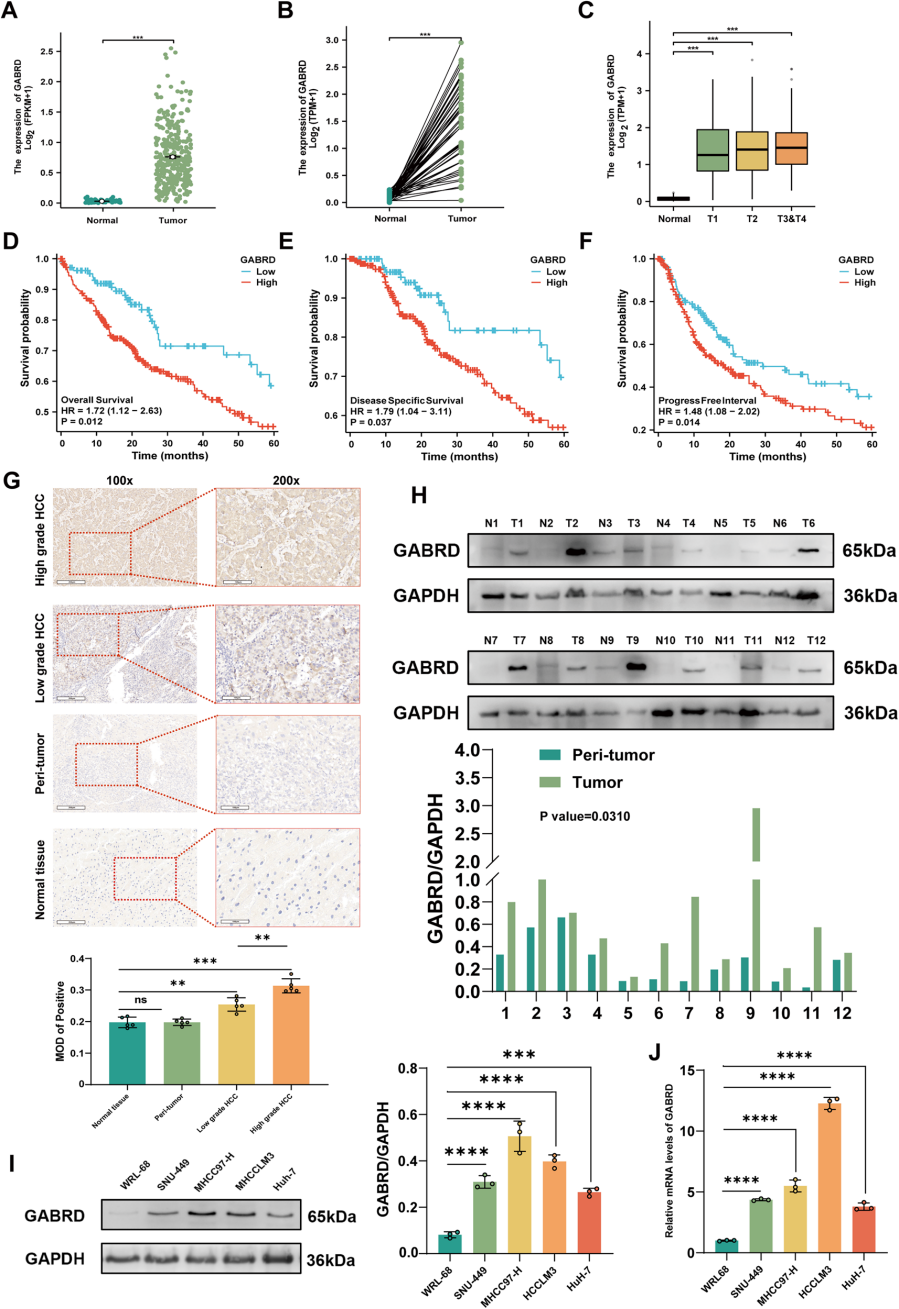
GABRD is upregulated in HCC and correlates with poor prognosis. **A** Grouped scatter plot comparing mRNA expression of GABRD (log_2_(FPKM + 1)) between HCC tissues and adjacent normal tissues from TCGA database. **B** Paired scatter plot showing GABRD expression (log_2_(TPM + 1)) in matched normal-tumor tissue pairs from the TCGA database. **C** Box plot illustrating GABRD expression (log_2_(TPM + 1)) across normal tissues and HCC tissues of different stages (T1, T2, T3, and T4). **D**–**F** Kaplan–Meier survival analysis of HCC patients stratified by GABRD expression: Overall survival (**D**), disease-specific survival (**E**), progression-free interval (**F**). **G** Immunohistochemical staining of GABRD protein in HCC tissue microarrays, comparing normal tissues, peri-tumor, low-grade HCC, and high-grade HCC. Scale bars: 200 µm (left) and 100 μm (right), *n* = 5. **H** Immunoblotting of GABRD protein expression in 12 paired HCC and adjacent tissues. **I**, **J** Immunoblotting (**I**) and qPCR analysis (**J**) of GABRD protein and mRNA expression in HCC cell lines (SNU449, MHCC97H, HCCLM3, and HuH7) vs. normal hepatocyte line WRL68, *n* = 3. Data were presented as mean ± SD. Statistical significance was determined by two-tailed Student’s *t*-test (**A**, **B**, **H**) and one-way ANOVA (**C**, **G**, **I**, **J**). Survival curves (**D**, **E**, **F**) were compared using the log-rank test. *P < 0. 05, **P < 0. 01, ***P < 0. 001, ****P < 0. 0001 and ns no significance.

To investigate the clinical significance of GABRD in HCC, we analyzed the TCGA-LIHC database, which revealed that GABRD mRNA was significantly upregulated in HCC tissues compared with adjacent normal tissues (Fig. 1A–C). Furthermore, high GABRD expression was significantly associated with poorer overall survival (OS), disease-specific survival (DSS), and progression-free interval (PFI) in HCC patients (Fig. 1D–F). External validation in the GEO dataset with the same grouping method confirmed that high GABRD expression was also significantly correlated with shorter overall survival in HCC patients, consistent with the results from the TCGA database (Fig. S1A).

Consistent with these findings, immunohistochemical (IHC) analysis of tissue microarrays demonstrated that GABRD protein levels were elevated in HCC tissues relative to adjacent normal tissues, with high-grade tumors showing even greater expression than low-grade tumors (Fig. 1G). This upregulation was further confirmed at the protein level by Western blot analysis of 12 paired HCC and adjacent tissue samples, which revealed higher GABRD expression in tumor lesions (Fig. 1H). Similarly, in vitro analysis showed that both mRNA and protein expression of GABRD were markedly elevated in a panel of HCC cell lines (MHCC97H, HCCLM3, HuH7, and SNU449) compared to the normal hepatocyte line WRL68 (Fig. 1I, J).

Collectively, these findings demonstrate that GABRD is consistently upregulated in HCC at both the transcriptional and protein levels, and its high expression correlates with poor HCC patient prognosis.

### GABRD knockout suppresses the proliferation, invasion, and migration of HCC cells

Based on our initial expression profiling, we selected the MHCC97H and HCCLM3 cell lines, which exhibit relatively high GABRD expression, to generate stable GABRD-knockout (GABRD-KO) cell lines, using a non-targeting control (NTC) as the negative control (Figs. S2A–D and S7A).

**Fig. 2:**
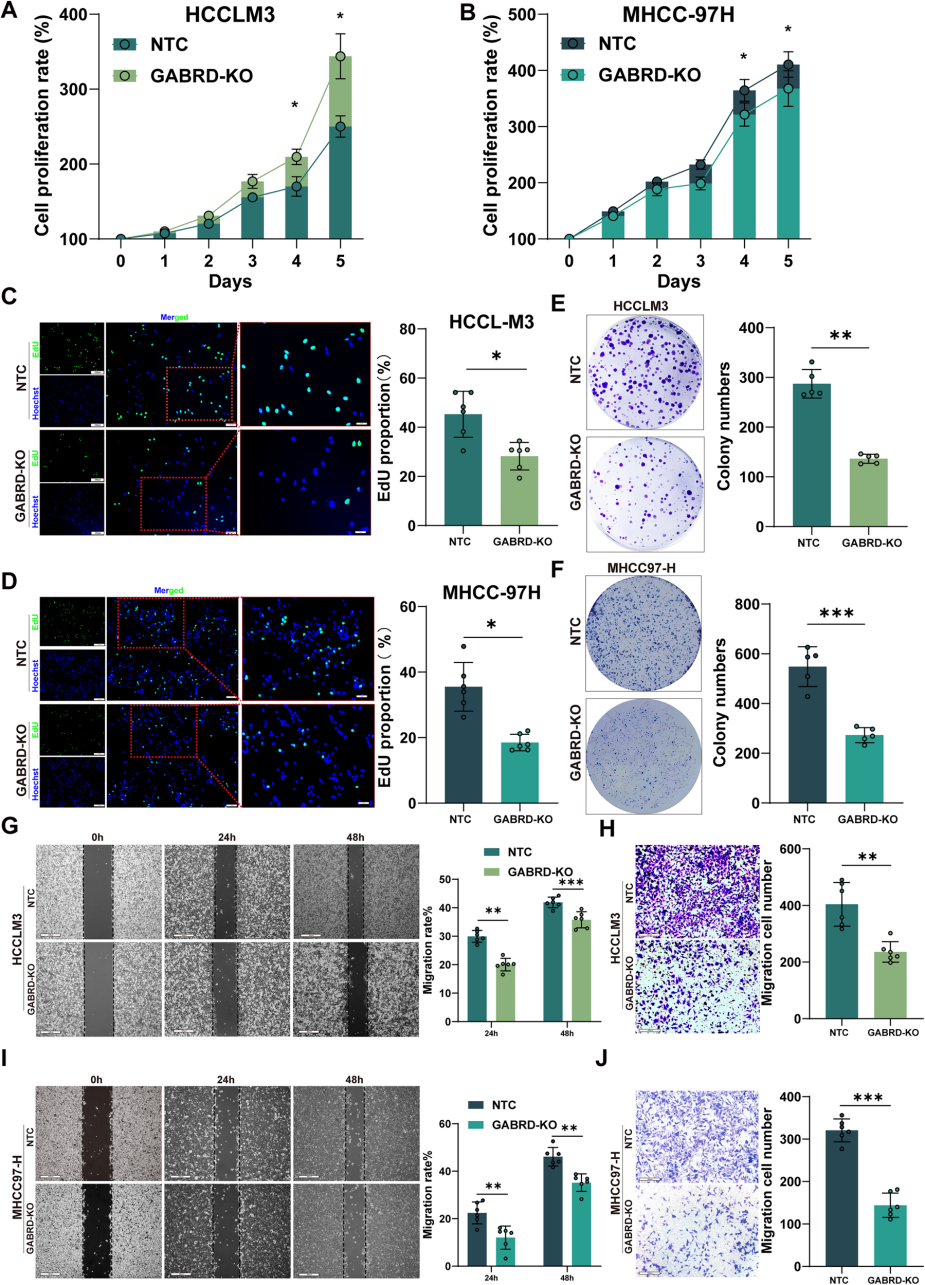
GABRD knockout suppresses the proliferation, invasion, and migration of HCC cells. **A**, **B**. Cell Counting Kit-8(CCK-8) showing proliferation rate of HCCLM3 (**A**) and MHCC97H (**B**) cells transfected with GABRD-KO and NTC at 0, 24, 48, 72, 96, and 120 h, *n* = 5. **C**, **D**. EdU fluorescence labeling and quantification of EdU-positive cells in GABRD-KO and NTC groups of HCCLM3 (**C**) and MHCC97H (**D**) cells, *n* = 6, Scale bar: 200 µm (left), 100 μm (middle) and 50 μm (right). **E**, **F**. Colony formation assays and quantification of clone numbers in GABRD-KO and NTC groups of HCCLM3 (**E**) and MHCC97H (**F**) cells, *n* = 5. **G**, **I**. Wound healing assays and migration rates in GABRD-KO and NTC cells at 24 and 48 h of HCCLM3 (**G**) and MHCC97H (**I**), *n* = 6, Scale bar: 500 µm. **H**, **J**. Transwell invasion assays and quantification of invaded cells in GABRD-KO and NTC groups of HCCLM3 (**H**) and MHCC97H (**J**) cells, *n* = 6, Scale bar: 200 µm. Data were presented as mean ± SD. Statistical significance was determined by two-tailed Student’s *t*-test (**C**–**F**, **H**, **J**) and two-way ANOVA with Bonferroni post hoc test (**A**, **B**, **G**, **I**). *P < 0. 05, **P < 0. 01, ***P < 0. 001, ****P < 0. 0001 and ns no significance.

To assess the impact of GABRD on cell proliferation, we performed CCK-8 assays, which revealed a significant reduction in the cell proliferation rate of GABRD-KO-transfected MHCC97H and HCCLM3 cells at 96 and 120 h post-seeding, indicating a slower proliferation rate (Fig. 2A, B). This finding was further corroborated by EdU incorporation assays, which showed a markedly lower percentage of EdU-positive cells in the GABRD-KO group (Fig. 2C, D). Furthermore, colony formation assays demonstrated that the number of colonies were substantially decreased upon GABRD knockout, reflecting an impaired clonogenic capacity (Fig. 2E, F).

To evaluate migration and invasion, wound healing assays showed that the wound closure rate was significantly slower in GABRD-KO-transfected cells compared to NTC controls at 24 and 48 h (Fig. 2G, I). Consistent with this, Transwell assays revealed a dramatic decrease in the number of cells traversing the membrane after GABRD knockout, confirming its role in suppressing the migratory and invasive potential of HCC cells (Fig. 2H, J). Collectively, these results indicate that GABRD knockout attenuates the malignant phenotypes of HCC cells in vitro.

### GABRD overexpression promotes the proliferation, invasion, and migration of HCC cells

**Fig. 3:**
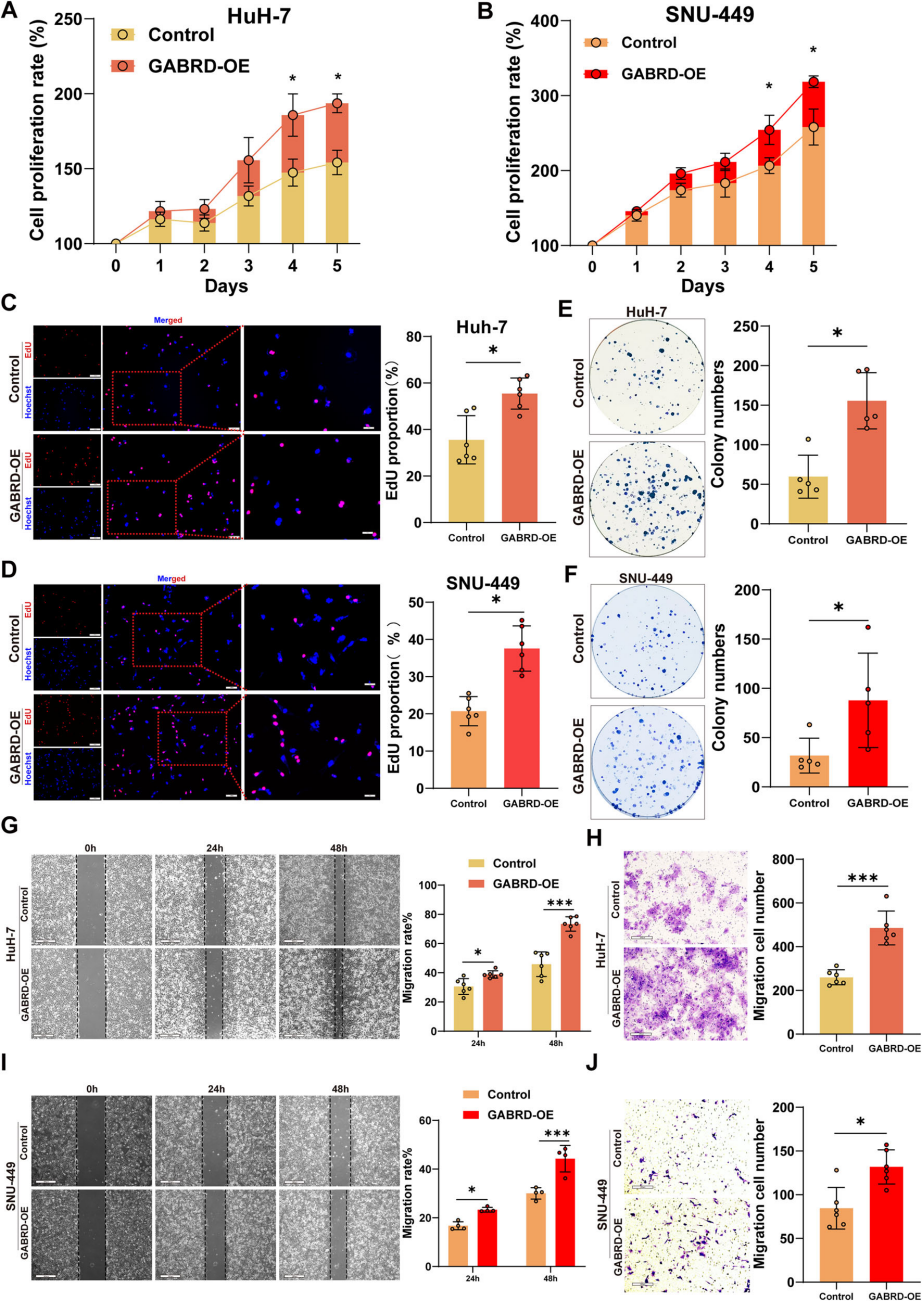
GABRD overexpression promotes proliferation, invasion, and migration of HCC cells. **A**, **B** CCK-8 showing proliferation rate in GABRD-OE and control of HuH-7 (**A**) and SNU449 (**B**) cells at 0, 24, 48, 72, 96, and 120 h, *n* = 5. **C**, **D** EdU fluorescence labeling and quantification of EdU-positive cells in GABRD-OE and control groups of HuH-7 (**C**) and SNU449 (**D**) cells, *n* = 6. Scale bar: 200 µm (left), 100 μm (middle), and 50 μm (right). **E**, **F** Colony formation assays and quantification of clone numbers in GABRD-OE and control groups of HuH-7 (**E**) and SNU449 (**F**) cells, *n* = 5. **G**, **I** Wound healing assays and migration rates in GABRD-OE and control cells at 24 and 48 h of HuH-7 (**G**) and SNU449 (**I**), *n* = 6. Scale bar: 500 µm. **H**, **J** Transwell invasion assays and quantification of invaded cells in GABRD-OE and control groups of HuH-7 (**H**) and SNU449 (**J**) cells, *n* = 6. Scale bar: 200 µm. Data were presented as mean ± SD. Statistical significance was determined by two-tailed Student’s *t*-test (**C**–**F**, **H**, **J**) and two-way ANOVA with Bonferroni post hoc test (**A**, **B**, **G**, **I**). *P < 0. 05, **P < 0. 01, ***P < 0. 001, ****P < 0. 0001, and ns no significance.

To complement these loss-of-function studies, we performed gain-of-function assays by overexpressing GABRD in the HuH7 and SNU449 cell lines, which have relatively low endogenous GABRD levels, using an empty vector (Control) as a reference (Fig. S1E–H). GABRD overexpression significantly enhanced proliferative capacity, as demonstrated by increased cell proliferation rate in CCK-8 assays (Fig. 3A, B), a higher percentage of EdU-positive cells (Fig. 3C, D), and increased colony formation (Fig. 3E, F). In line with these findings, migration and invasion assays revealed that GABRD-overexpressing cells exhibited accelerated wound closure (Fig. 3G, I) and a greater ability to penetrate Transwell membranes (Fig. 3H, J). These gain-of-function data confirm that GABRD acts as a potent promoter of HCC cell aggressiveness.

### GABRD promotes HCC proliferation in vivo

**Fig. 4:**
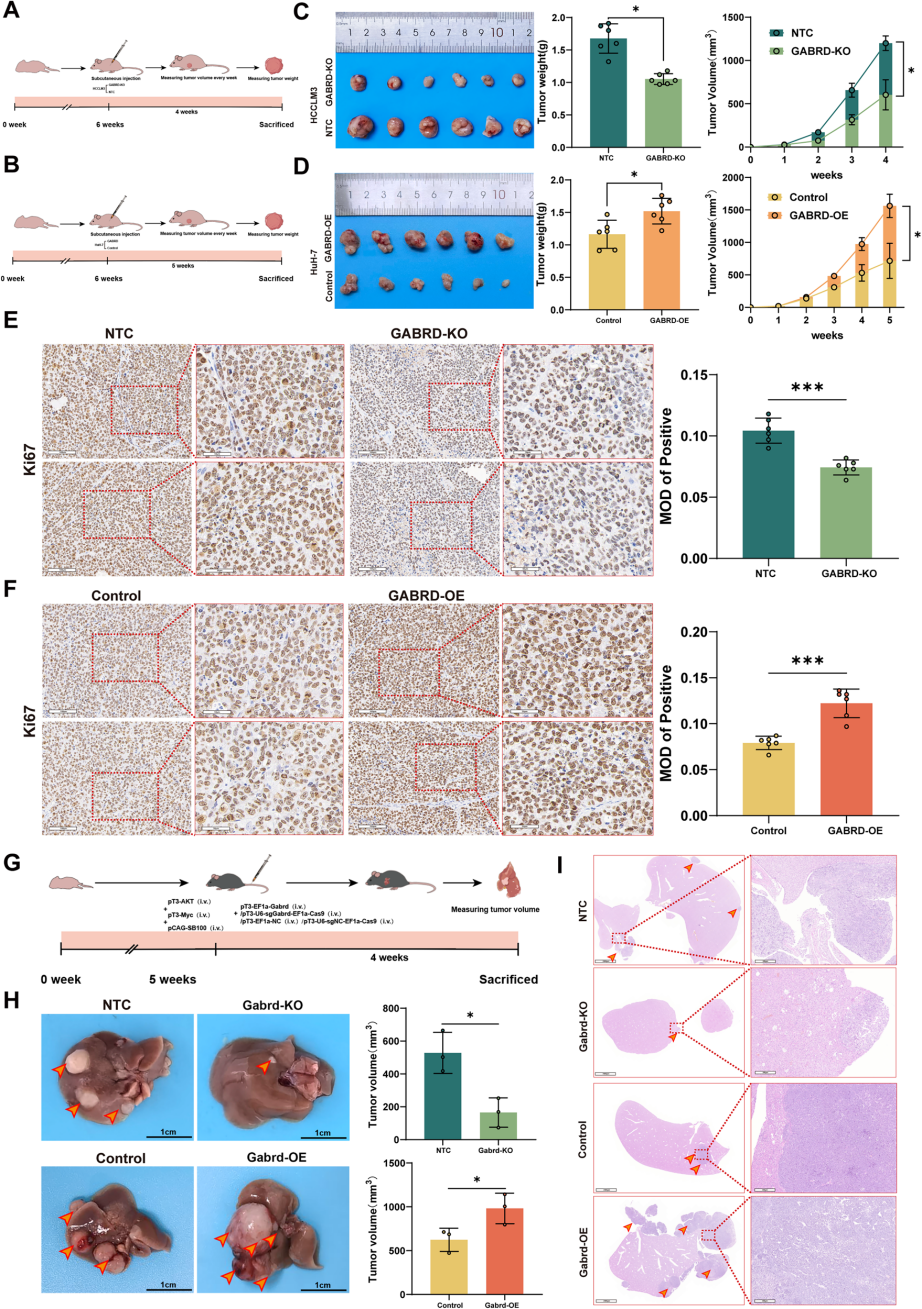
GABRD promotes HCC proliferation in *vivo.* **A** Schematic of the subcutaneous xenograft model using GABRD-KO-HCCLM3 and NTC-HCCLM3. **B** Schematic of the subcutaneous xenograft model using GABRD-OE-HuH-7 and Control-HuH-7. **C** Representative images and quantification of tumor weight and volume in GABRD-KO vs. NTC, *n* = 6. **D** Representative images and quantification of tumor weight and volume in GABRD-OE vs. Control, *n* = 6. **E**, **F** Immunohistochemical staining and mean optical density (MOD) of Ki-67 in GABRD-KO vs. NTC (**E**) and GABRD-OE vs. Control (**F**) tumor tissues, *n* = 6. Scale bar: 100 µm (left) and 50 μm (right). **G** Schematic of hydrodynamic transfection-induced spontaneous HCC model via Myc/AKT co-expression with subsequent tail vein injection of Gabrd-modifying plasmids. **H** Representative liver images and quantification of tumor volume in Gabrd-KO, NTC, Gabrd-OE, and Control mice, *n* = 3. **I** Hematoxylin and eosin (HE) staining of liver in spontaneous HCC mice. Scale bar: 2000 µm (left) and 200 µm (right). Statistical significance was determined by two-tailed Student’s *t*-test (**C**–**F**) and one-way ANOVA with Tukey’s post hoc test (**H**). Growth curves were analyzed by two-way ANOVA with Bonferroni post hoc test, *P < 0. 05, **P < 0. 01, ***P < 0. 001 ****P < 0. 0001, and ns no significance.

To investigate the role of GABRD in HCC progression in *vivo*, we employed two complementary animal models. First, we established a subcutaneous xenograft model using GABRD-knockout HCCLM3 cells (GABRD-KO) and their corresponding controls (NTC), as well as GABRD-overexpressing HuH7 cells (GABRD-OE) and vector controls (Control) (Fig. 4A, B). Longitudinal monitoring revealed that tumor growth was significantly attenuated in the GABRD-KO group compared to the NTC group. Conversely, tumors derived from GABRD-OE cells grew markedly faster than those from the Control group (Fig. 4C, D). Consistent with these growth kinetics, IHC analysis of GABRD and the proliferation marker Ki-67 showed a significantly lower percentage of Ki-67-positive cells in GABRD-KO tumors and a higher percentage in GABRD-OE tumors compared to their respective controls (Fig. 4E, F and Fig. S3A, B).

To validate these findings in a more physiologically relevant context, we utilized a hydrodynamic tail vein injection (HTVI) model to induce spontaneous HCC formation in mice by co-expressing the Myc and AKT oncogenes (Fig. 4G). Gross examination of livers from Gabrd-knockout (Gabrd-KO) mice revealed a significant reduction in both tumor number and volume compared to non-targeting controls. In contrast, Gabrd-overexpressing (Gabrd-OE) mice developed significantly larger and more numerous tumor nodules (Fig. 4H, I and Fig. S3C, D). Taken together, these results from both xenograft and genetically engineered mouse models provide robust evidence that GABRD functions as a critical promoter of HCC proliferation in vivo.

### GABRD drives HCC progression via EMT and JAK2-STAT3 activation

**Fig. 5:**
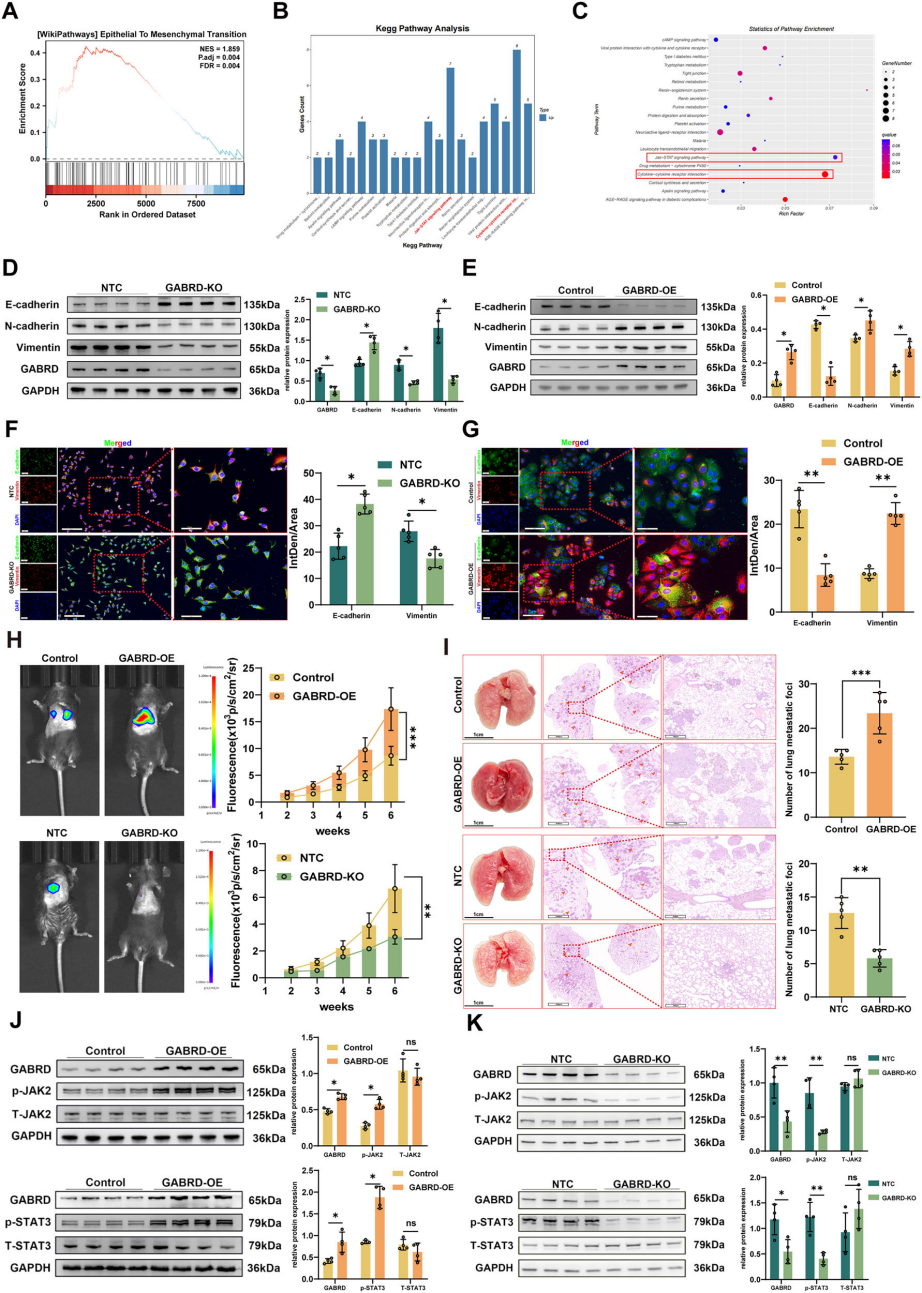
GABRD drives HCC progression via EMT and JAK2-STAT3 activation. **A** GSEA plot showing enrichment of EMT-related gene sets in GABRD-OE HuH7 cells. **B**, **C** KEGG pathway enrichment analysis highlighting cytokine–cytokine receptor interaction and JAK-STAT signaling in GABRD-OE cells. **D**, **E** Immunoblotting and quantification of EMT markers in (**D**) GABRD-KO-HCCLM3 and (**E**) GABRD-OE-HuH7 cells, *n* = 4. **F**, **G** Immunofluorescence staining of E-cadherin and Vimentin in (**F**) GABRD-KO-HCCLM3 and (**G**) GABRD-OE-HuH7 cells, *n* = 5, Scale bar: 200 µm (left), 200 μm (middle), and 100 μm (right). **H** Bioluminescence imaging of mice at 6 weeks after implantation, including Gabrd-OE, Control, Gabrd-KO, and NTC group. Bioluminescent signal intensity reflects the quantity and activity of implanted cells, *n* = 5. **I** HE staining of the lung in mice and quantification of metastatic nodules. Scale bar: 2000 µm (left) and 200 µm (right), *n* = 5. **J**, **K** Immunoblotting and quantification of phosphorylated (p-)JAK2, total JAK2, p-STAT3, and total STAT3 in (**J**) GABRD-OE-HuH7 and (**K**) GABRD-KO-HCCLM3 cells, *n* = 4. Statistical significance was determined by a two-tailed Student’s *t*-test. *P < 0. 05, **P < 0. 01, ***P < 0. 001, ****P < 0. 0001, and ns no significance.

To clarify the molecular mechanisms underlying GABRD-mediated HCC progression, we performed RNA sequencing (RNA-seq) on GABRD-overexpressing HuH-7 cells and vector controls (Fig. S4A, B). Gene set enrichment analysis (GSEA) revealed a significant enrichment of gene sets associated with epithelial-mesenchymal transition (EMT) in GABRD-overexpressing cells (Fig. 5A), suggesting that GABRD may regulate the metastatic potential of HCC cells through EMT induction.

Given that EMT is a critical process in cancer metastasis and is often regulated by cytokine signaling pathways, we further analyzed the KEGG pathway enrichment of differentially expressed genes. Notably, the cytokine–cytokine receptor interaction pathway and the JAK-STAT signaling pathway were among the most significantly enriched pathways in the GABRD overexpression group (Fig. 5B, C). The enrichment of these pathways is particularly relevant because JAK-STAT signaling is a well-established regulator of EMT in various cancers [22, 23], while GABRD is a membrane protein, suggesting it may exert its effects through membrane receptor-mediated signaling, and the cytokine–cytokine receptor interaction pathway enrichment further points to potential membrane receptor involvement in GABRD’s oncogenic mechanism. Western blot analysis confirmed this. GABRD knockout in HCCLM3 cells led to the upregulation of the epithelial marker E-cadherin and the downregulation of the mesenchymal markers N-cadherin and Vimentin (Fig. 5D). Reciprocally, GABRD overexpression in HuH-7 cells induced the opposite protein expression pattern (Fig. 5E), and these findings were further corroborated by immunofluorescence staining (Fig. 5F, G). To assess the functional consequences of this EMT induction in vivo, we established a lung metastasis model via tail vein injection of Gabrd-OE/KO Hepa1-6 cells. Six weeks after tail vein injection of cells, IVIS imaging showed that the total fluorescence signal intensity in the lungs of mice in the Gabrd-overexpressing group was significantly higher than that in the Control group (Fig. 5H), indicating that more HCC cells successfully colonized and proliferated in the lungs. In contrast, the pulmonary fluorescence signal in the Gabrd-knockout group was significantly weaker than that in the NTC group. Successful establishment of the model was verified by IHC of GABRD (Fig. S5A, B). Mouse lungs were harvested for HE staining, and metastatic nodules were counted under microscopy (Fig. 5I). The results demonstrated that the number of lung metastatic nodules was significantly higher in the Gabrd-overexpressing group than in the Control group, while significantly fewer metastatic nodules were observed in the Gabrd-knockout group compared with the NTC group. Immunohistochemical staining of the tissue sections for EMT markers showed that the Gabrd-knockout group exhibited elevated staining intensity of E-cadherin and decreased expression of Vimentin. Conversely, the Gabrd-overexpressing group displayed reduced staining intensity of E-cadherin and enhanced expression of Vimentin (Fig. S5C–F). This histological evidence was highly consistent with the in vivo bioluminescence imaging results, collectively confirming that GABRD significantly promotes the survival and colonization capacity of HCC cells in distant organs by inducing an epithelial-mesenchymal transition phenotype.

Given the established role of cytokine signaling in activating the JAK-STAT pathway to drive tumor progression and immune evasion [24, 25], and the significant enrichment of both cytokine–cytokine receptor interaction and JAK-STAT signaling pathways in our RNA-seq analysis, we hypothesized that GABRD might exert its oncogenic effects through the above axis. To test this, we examined the phosphorylation status of key JAK-STAT components (Fig. S5G, H). Western blot analysis revealed that GABRD overexpression significantly increased the phosphorylation levels of JAK2 and STAT3, without affecting their total protein levels (Fig. 5J). Conversely, GABRD knockout led to a marked decrease in the phosphorylation of JAK2 and STAT3 (Fig. 5K). Collectively, these findings demonstrate that GABRD drives HCC progression by orchestrating the activation of EMT and the JAK2-STAT3 pathway.

### JAK2-STAT3 inhibition reverses GABRD-mediated oncogenic and metastatic effects in HCC

**Fig. 6:**
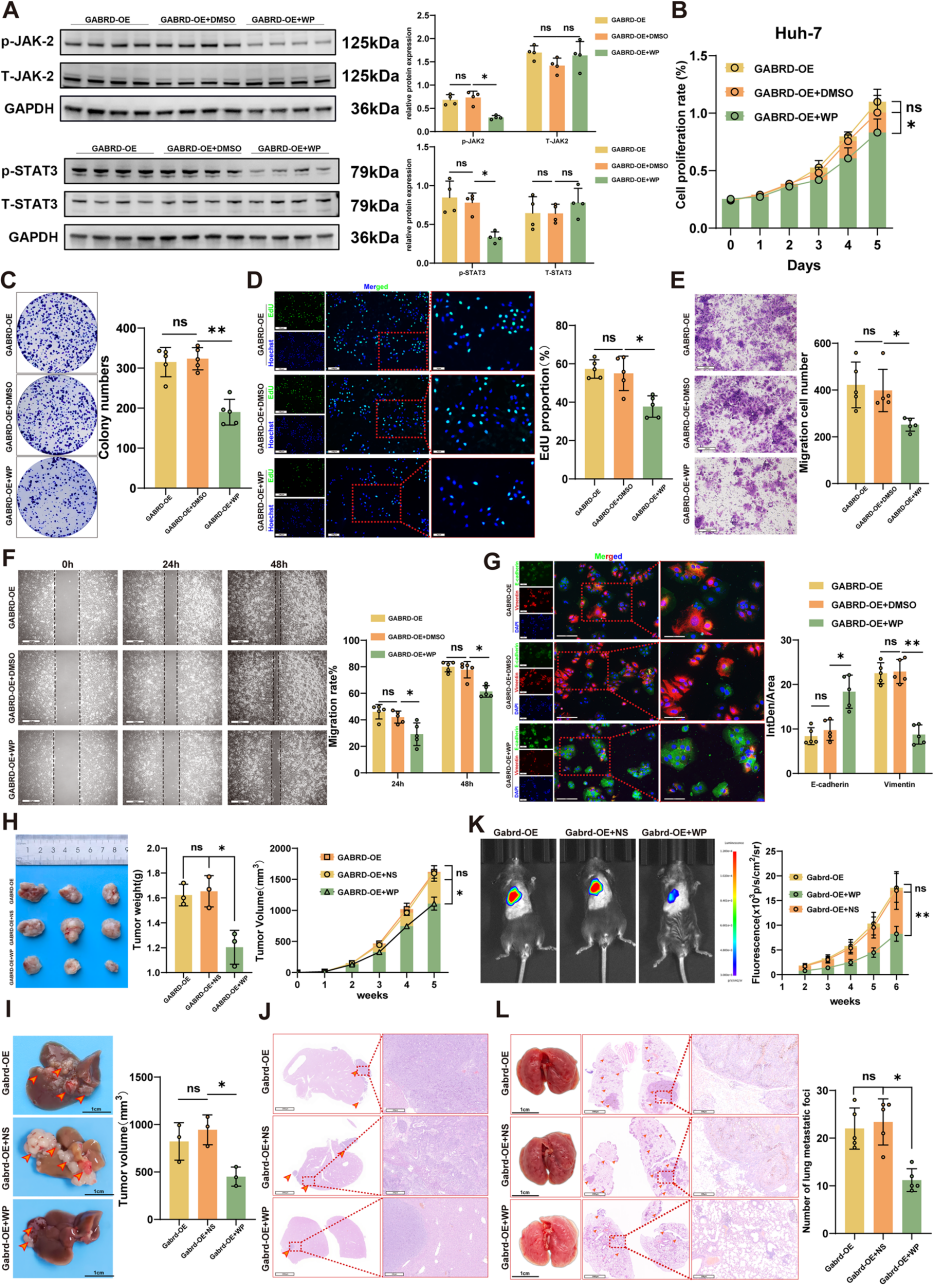
JAK2-STAT3 inhibition reverses GABRD-mediated oncogenic effects in HCC. **A** Immunoblotting and quantification of phosphorylated and total JAK2/STAT3 in GABRD-OE-HuH-7 cells treated with WP1066, DMSO and control, *n* = 4. **B** CCK-8 assay showing cell proliferation rate with WP1066, DMSO and control in GABRD-OE cells, *n* = 5. **C** Colony formation assays and quantification of clone numbers in WP1066, DMSO and control groups, *n* = 5. **D** EdU assay and quantification of EdU-positive cells, *n* = 5, Scale bar: 200 µm (left), 100 μm (middle), and 50 μm (right). **E** Transwell invasion assay and corresponding quantification in the WP1066, DMSO and control groups, *n* = 5. Scale bar: 200 µm. **F** Wound healing assay and corresponding quantification in the WP1066, DMSO and control groups, *n* = 5. Scale bar: 500 µm. **G** Immunofluorescence staining of E-cadherin and Vimentin treated with WP1066, DMSO and control in GABRD-OE-HuH7 cells, *n* = 5, Scale bar: 200 µm (left), 200 μm (middle), and 100 μm (right). **H** Tumor volumes, weights, and images from subcutaneous xenografts of GABRD-OE HuH-7 cells with WP1066, normal saline (NS) and control treatment, *n* = 3. **I**, **J** Representative liver images and HE staining from spontaneous HCC model with WP1066, NS and control treatment, *n* = 3. Scale bar: 1 cm (left), 2000 µm (middle), and 200 µm (right). **K** In vivo bioluminescence imaging at 6 weeks after implantation in a lung metastasis model, including GABRD-OE group, GABRD-OE + NS group, and GABRD-OE + WP1066 group. Bioluminescent signal intensity reflects the quantity and activity of implanted cells, *n* = 5. **L** HE staining of lung in mice and quantification of metastatic nodules, *n* = 5, Scale bar: 1 cm (left), 2000 µm (middle), and 200 µm (right). Statistical significance was determined by one-way ANOVA with Tukey’s post hoc test (**A**, **C**, **D**, **E**, **G**–**I**, **L**), CCK-8, wound healing and growth curves were analyzed by two-way ANOVA with Bonferroni post hoc test(**B**, **F**, **H**, **K**). *P < 0. 05, **P < 0. 01, ***P < 0. 001, ****P < 0. 0001, and ns no significance.

To test the functional reliance of GABRD’s oncogenic activity on JAK2-STAT3 signaling, we treated GABRD-overexpressing HuH-7 cells with the specific JAK2-STAT3 inhibitor WP1066 (Fig. 6A). In *vitro*, WP1066 treatment significantly blunted the pro-proliferative effects of GABRD overexpression, as evidenced by reduced cell viability in CCK-8 assays (Fig. 6B), diminished colony formation (Fig. 6C), and decreased EdU incorporation (Fig. 6D). Likewise, the enhanced invasive and migratory capacities conferred by GABRD were effectively suppressed by WP1066 treatment (Fig. 6E, F). Furthermore, western blot analysis confirmed that following inhibition of the JAK2/STAT3 pathway, the changes in EMT-related markers induced by GABRD overexpression were reversed: E-cadherin was significantly increased, whereas Vimentin was markedly decreased and restored to baseline levels in GABRD-OE cells after inhibitor treatment (Fig. S6A). Consistent with these functional assays, immunofluorescence analysis confirmed that WP1066 treatment reversed the changes in EMT-related markers induced by GABRD overexpression (Fig. 6G).

We next validated these findings in vivo. In mice bearing GABRD-overexpressing HuH-7 xenografts, WP1066 administration led to a marked reduction in tumor volume and weight compared to saline-treated controls (Fig. 6H). This antitumor effect was corroborated by IHC analysis, which revealed a significant decrease in Ki-67 positivity in the WP1066-treated group (Fig. S6B). Consistent with these results, in the more physiologically relevant Myc/AKT-induced spontaneous HCC model, WP1066 treatment also significantly reduced the tumor burden in Gabrd-OE mice (Fig. 6I, J). Furthermore, in a lung metastasis mouse model, the pro-metastatic effect of GABRD overexpression was abrogated by WP1066 treatment (Fig. 6K, L). Collectively, these findings establish that GABRD exerts oncogenic and metastatic effects on HCC via activation of the JAK2-STAT3 signaling pathway.

### GABRD promotes HCC progression by binding to IL-10RA

**Fig. 7:**
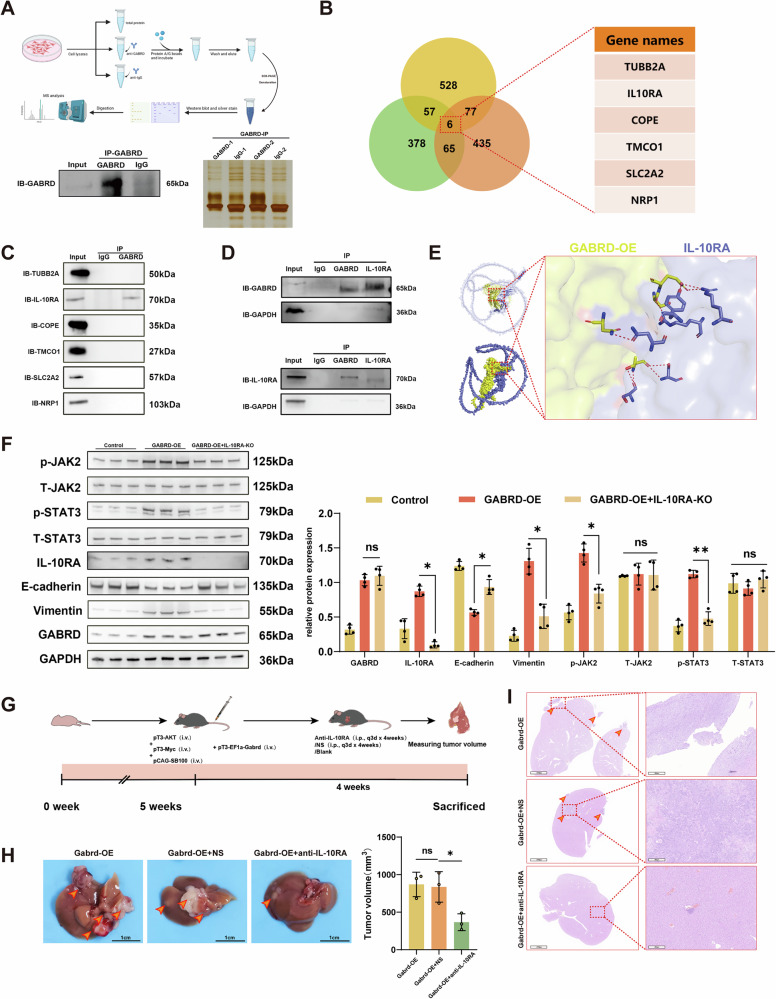
GABRD promotes HCC progression by binding to IL-10RA. **A** Schematic of Co-IP coupled with mass spectrometry (MS) used to screen for GABRD-interacting proteins, showing GABRD immunoblot and silver staining of proteins immunoprecipitated by a GABRD antibody. **B** Venn diagram of candidate interacting proteins from the MS analysis. **C** Immunoblot validation of top candidate interacting proteins from the GABRD co-immunoprecipitation. **D** Reciprocal Co-IP validating GABRD/IL-10RA interaction in GABRD-OE-HuH7 cells. **E** Molecular docking analysis predicting the binding interface between the GABRD and IL-10RA proteins. **F** Immunoblotting and quantification of p-JAK2, p-STAT3, T-JAK2, T-STAT3, and EMT markers including E-cadherin and Vimentin in Control-HuH7, GABRD-OE-HuH7 and GABRD-OE-HuH7 cells with IL-10RA knockout, *n* = 4. **G** Schematic of IL-10RA neutralizing antibody treatment(40 mg/kg, intraperitoneal injection twice weekly) in Myc/AKT-induced mice. **H**, **I** Representative liver images and HE staining from spontaneous HCC model with IL-10RA neutralizing antibody, NS and control treatment, *n* = 3. Scale bar: 1 cm (left), 2000 µm (middle), and 200 µm (right). Statistical significance was determined by a two-tailed Student’s *t*-test. *P < 0. 05, **P < 0. 01, ***P < 0. 001, ****P < 0. 0001, and ns no significance.

Building upon our prior findings implicating the activation of JAK2-STAT3 and cytokine–cytokine receptor signaling pathways, we aimed to identify the direct molecular mediator linking GABRD to the activated JAK2-STAT3 and cytokine–cytokine receptor pathways. As GABRD is a membrane protein, we employed co-immunoprecipitation (Co-IP) coupled with mass spectrometry to identify its binding partners (Fig. 7A). This analysis revealed several candidate proteins, including TUBB2A, IL-10RA, COPE, TMCO1, SLC2A2, and NRP1 (Fig. 7B).

We then systematically validated these candidates using individual Co-IP assays. Among them, only IL-10RA co-immunoprecipitated with GABRD, revealing a specific physical interaction (Fig. 7C). This interaction was further confirmed by reciprocal Co-IP assays (Fig. 7D). To provide structural insight, we performed molecular docking analysis, which predicted a direct binding interface between GABRD and IL-10RA (Fig. 7E). This in silico evidence corroborated our experimental data, strengthening the case for a direct interaction.

The identification of IL-10RA as a GABRD partner is particularly noteworthy. IL-10RA is a critical subunit of the IL-10 receptor complex, which, upon heterodimerization with IL-10RB, activates downstream JAK kinases that phosphorylate STAT3 [26, 27]. This established signaling cascade directly connects our findings to the JAK2-STAT3 pathway, whose activation we had previously linked to GABRD. Therefore, we hypothesized that the GABRD-IL-10RA interaction serves as the molecular conduit for GABRD-mediated activation of the JAK2-STAT3 axis.

To functionally validate the importance of the GABRD-IL-10RA axis, we performed IL-10RA knockout experiments in GABRD-overexpressing HCC cells. Western blot analysis demonstrated that IL-10RA knockout significantly attenuated the activation of JAK2/STAT3 signaling (reduced p-JAK2 and p-STAT3 levels) and reversed EMT marker changes induced by GABRD overexpression (Fig. 7F).

In vivo, we treated Gabrd-OE mice in the Myc/AKT HCC model with a neutralizing IL-10RA antibody (Fig. 7G). Strikingly, blockade of IL-10RA significantly reduced the volume of tumor nodules compared to isotype control-treated mice (Fig. 7H, I). Western blot analysis of tumor tissues revealed that the neutralizing antibody significantly reversed the activation of p-JAK2 and p-STAT3 in tumors induced by GABRD overexpression (Fig. S7A). This result provides compelling in vivo evidence that the oncogenic effects of GABRD are mediated through its interaction with IL-10RA.

Furthermore, we investigated whether GABRD and IL-10RA reciprocally regulate each other’s expression. Our data demonstrated that GABRD knockout led to a significant decrease in IL-10RA protein expression, whereas IL-10RA mRNA levels remained unchanged (Fig. S7B). In contrast, knockout of IL-10RA had no impact on GABRD expression (Fig. S7C). These findings suggest that GABRD may regulate IL-10RA expression at the post-translational level, potentially by modulating protein stability or influencing degradation pathways. In summary, our data delineate a novel oncogenic mechanism in HCC: GABRD physically interacts with IL-10RA to form a receptor complex, which activates the JAK2-STAT3 signaling axis and thereby drives tumor progression (Fig. S7D).

## Materials and methods

### Clinical specimens

HCC tissue microarrays were purchased from (servicebio), which contained HCC tissues and adjacent normal tissues, as well as HCC tissues of different grades (high-grade and low-grade). Twelve pairs of tissue specimens were collected from HCC patients undergoing surgical resection at Xinqiao Hospital, with exclusion criteria applied to individuals who had received chemotherapy or radiotherapy. All tissue samples were confirmed by pathological diagnosis, and the collection of samples was approved by the Medical Ethics Committee of Second Affiliated Hospital of Army Medical University, PLA.(2020-YD081-01) Informed consent was obtained from all patients.

### Database source and analysis

The mRNA expression data of GABRD in HCC tissues and adjacent normal tissues, along with corresponding clinical survival information, were retrieved from The Cancer Genome Atlas-Liver Hepatocellular Carcinoma (TCGA-LIHC) database. Differential expression analysis of GABRD between tumor and normal tissues was performed using R. For survival analysis, the cut‑off value for GABRD expression was defined as the arithmetic mean of log2(TPM + 1)-normalized GABRD mRNA expression values in the entire HCC cohort. Patients with GABRD expression levels above the mean value were categorized into the high-expression group, and those with levels below the mean value were classified into the low-expression group. Kaplan‑Meier curves and log‑rank test were used to compare overall survival (OS), disease‑specific survival (DSS), and progression‑free interval (PFI) between the two groups. For external validation, the same grouping and statistical methods were applied to an independent HCC cohort from the Gene Expression Omnibus (GEO) dataset.

### Cell culture

The HCC cell lines MHCC97H, HCCLM3, HuH7, SNU449, and Hepa1-6, along with the normal hepatocyte line WRL68, were obtained from the Shanghai Cell Bank Type Culture Collection Committee. MHCC97H, HCCLM3, HuH7, and Hepa1-6 cells were cultured in Dulbecco’s modified Eagle’s medium (DMEM) supplemented with 10% fetal bovine serum (FBS, Gibco, USA), and SNU449 cells were maintained in RPMI-1640 supplemented with 10% FBS. All cells were cultured at 37 °C in a humidified incubator containing 5% CO_2_.

### Cell transfection and stable cell line establishment

To establish stable GABRD-knockout human HCC cell lines, lentiviral vectors encoding puromycin resistance and the CRISPR-Cas9 system were generated. Knockout was achieved using a lentiCRISPR v2 backbone co-expressing SpCas9 and a validated human GABRD-targeting single-guide RNA (sgRNA). For overexpression, a pLVX-IRES-Puro vector carrying the human GABRD cDNA driven by a CMV promoter was used. Lentiviruses were produced by co-transfecting HEK293T cells with the transfer vectors and packaging plasmids (psPAX2, pMD2-G) using Lipofectamine 3000. Supernatants harvested at 48 and 72 h post-transfection were concentrated via PEG-it. Human HCC cells were transduced with the virus in the presence of 8 μg/mL polybrene for 24 h, followed by selection with 2 μg/mL puromycin for 7 days. Validation was conducted using qRT-PCR and Western blotting to confirm the absence of GABRD protein. For stable GABRD-knockout Hepa1-6 cell lines, lentiviral vectors co-expressing firefly luciferase, puromycin resistance, and the CRISPR-Cas9 system were generated. Knockout employed a modified lentiCRISPR v2 vector expressing SpCas9, a validated murine Gabrd-targeting sgRNA, and an IRES-firefly luciferase cassette. For overexpression, pLVX-IRES-Puro harboring murine Gabrd cDNA (CMV-driven) was used. Lentivirus production, concentration, and cell transduction (using 8 μg/mL polybrene for 24 hours) were conducted as described previously. Stable populations were selected using 2 μg/mL puromycin over a period of 7 days. Validation encompassed luciferase activity assays, qRT-PCR, and Western blotting to confirm the loss of murine GABRD expression.

### Quantitative real-time PCR

Total RNA was extracted from cells using an RNA extraction kit (UE-MN-MS-RNA, UElandy). cDNA was synthesized with the Reverse Transcription Kit (FAQ-101, TOYOBO). qPCR was performed using a qPCR kit (G3326-15, Servicebio) on an ABI 7500 Real-Time PCR System. The thermocycling conditions were as follows: an initial denaturation at 95 °C for 30 s, followed by 40 cycles of denaturation at 95 °C for 5 s and annealing/extension at 60 °C for 30 s. A melting curve analysis was then performed to confirm product specificity. All reactions were performed in triplicate. GAPDH was used as the internal reference gene, and non-template controls (NTCs) were included in each run to monitor for contamination. Relative gene expression was calculated using the 2^−ΔΔCt^ method, normalizing GABRD expression to GAPDH. Data were presented as the mean ± standard deviation (SD) from at least three independent biological replicates.

### Western blot

Total protein was extracted from cells and tissues using RIPA lysis buffer (G2002; Servicebio) supplemented with protease and phosphatase inhibitors. Protein concentrations were determined using a BCA assay. Equal amounts of protein (e.g., 30 µg per lane) were separated by sodium dodecyl sulfate-polyacrylamide gel electrophoresis (SDS-PAGE) and transferred onto polyvinylidene difluoride (PVDF) membranes. The membranes were blocked in 5% non-fat dry milk in Tris-buffered saline with Tween-20 (TBST) for 1 h at room temperature and then incubated with primary antibodies overnight at 4 °C. After washing three times with TBST, the membranes were incubated with HRP-conjugated secondary antibodies for 1 h at room temperature. Signals were visualized using an enhanced chemiluminescence (ECL) substrate and a chemiluminescence imaging system.

### Immunohistochemistry

Formalin-fixed, paraffin-embedded (FFPE) HCC tissue microarrays and tumor samples were dewaxed in xylene and rehydrated through a graded ethanol series. Antigen retrieval was performed by heating the sections in citrate buffer (pH 6.0). Endogenous peroxidase activity was blocked with 3% hydrogen peroxide. Sections were then incubated with primary antibodies against GABRD and Ki-67 overnight at 4 °C, followed by incubation with an HRP-conjugated secondary antibody. Immunoreactivity was visualized using 3,3’-diaminobenzidine (DAB) as the chromogen, followed by counterstaining with hematoxylin. The immunostaining was quantified by calculating the H-score, which considers both staining intensity and the percentage of positive cells, using ImageJ software.

### Cell viability assay

Cells were seeded in 96-well plates at a density of 1 × 10³ cells/well. At 24, 48, 72, 96, and 120 h after seeding, 10 μL of CCK-8 solution was added to each well and incubated for 1 h at 37 °C. The absorbance at 450 nm was measured using a microplate reader.

### 5-Ethynyl-2’-deoxyuridine (EdU) incorporation assay

Cells were incubated with EdU solution for 2 hours at 37 °C. Following incubation, cells were fixed with 4% paraformaldehyde and permeabilized with 0.3% Triton X-100 in PBS. After permeabilization, the cells were stained with an Apollo reaction cocktail and counterstained with Hoechst 33342 to visualize nuclei. The percentage of EdU-positive cells was determined by analyzing at least five random fields per well under a fluorescence microscope.

### Clone formation assay

Cells were seeded in six-well plates at a low density of 500 cells per well and cultured for 10–14 days to allow for colony formation. The culture medium was replaced every 3–4 days. At the endpoint, colonies were fixed with 4% paraformaldehyde and stained with 0.5% crystal violet solution for 20 min. Colonies, defined as groups of ≥50 cells, were manually counted, and the results are presented as the mean number of colonies.

### Wound healing assay

Cells were seeded in six-well plates and grown to a confluent monolayer. A uniform scratch was made across the center of each well using a sterile 200 µL pipette tip. Cells were then washed twice with PBS to remove detached cells and cultured in serum-free medium. Images of the scratch were captured at 0, 24, and 48 h at the same location. The healing rate was calculated as [(initial wound width − final wound width)/initial wound width] × 100%.

### Transwell assay

Transwell chambers (8 µm pore size; Corning) were pre-coated with a diluted Matrigel matrix (1:8 in serum-free medium). A total of 5 × 10⁴ cells in 200 µL of serum-free medium were seeded into the upper chamber. The lower chamber was filled with 600 µL of complete medium containing 10% FBS as a chemoattractant. After 48 h of incubation, non-invaded cells on the upper surface of the membrane were gently removed with a cotton swab. Invaded cells on the lower surface were fixed with methanol, stained with crystal violet, and counted in five randomly selected fields under a microscope.

### RNA sequencing

RNA sequencing was performed by Sinotech Genomics. Total RNA was extracted from GABRD-overexpressing HuH-7 cells and vector control cells using TRIzol reagent. RNA integrity was assessed using an Agilent Bioanalyzer 2100, and purity and concentration were measured using a NanoDrop 2000 spectrophotometer. Only samples with RNA integrity number (RIN) >8 and A260/A280 ratio between 1.8 and 2.0 were used for library preparation. Stranded mRNA-seq libraries were prepared from 1 µg of total RNA using the Illumina TruSeq Stranded mRNA Library Prep Kit, which involved poly(A) selection for mRNA enrichment. The libraries were quantified by qPCR using the KAPA Library Quantification Kit and sequenced on an Illumina NovaSeq 6000 platform to generate paired-end 150 bp reads with a sequencing depth of ~40 million reads per sample. Raw sequencing data underwent quality control using FastQC v0.11.9, followed by adapter trimming and low-quality base removal using Trimmomatic v0.39 with parameters: LEADING:3, TRAILING:3, SLIDINGWINDOW:4:15, MINLEN:36. Clean reads were aligned to the human reference genome (GRCh38/hg38) using the STAR aligner v2.7.10a with default parameters. Gene-level expression quantification was performed using featureCounts v2.0.1 with the GENCODE v38 annotation file. Differential expression analysis was performed using DESeq2 v1.34.0 in R v4.1.2. Genes with |log2(fold change)| >1 and adjusted *p* value <0.05 were considered differentially expressed. Gene set enrichment analysis (GSEA) was performed using the clusterProfiler v4.2.2 package with the MSigDB hallmark gene sets. Kyoto Encyclopedia of Genes and Genomes (KEGG) pathway enrichment analysis was performed using clusterProfiler with a significance threshold of adjusted *p* value <0.05.

### Subcutaneous xenograft model

Nude mice (6 weeks old) were randomly divided into groups. HCCLM3-GABRD-KO, HCCLM3-NTC, HuH7-GABRD-OE, and HuH7-Control cells were suspended in 100 µL of a 1:1 mixture of serum-free medium and Matrigel and subcutaneously injected into the right flank of each mouse. Tumor volume was measured every 3 days using the formula: volume = (length×width²)/2. Mice were sacrificed after 4 weeks, and tumors were weighed and subjected to IHC analysis.

### Hydrodynamic transfection-induced spontaneous HCC model

C57BL/6 male mice, aged 5 weeks, were randomly assigned to four groups. To induce HCC, we performed tail vein hydrodynamic transfection of specific plasmid mixtures as previously described [28–30]. All four groups received 10 μg of pT3-AKT, 10 μg of pT3-Myc, and 1.25 μg of pCAG-SB100 as base plasmids. Additionally, each group was supplemented with 10 µg of a different target plasmid: the Gabrd-OE group received pT3-EF1a-Gabrd, the Gabrd-KO group received pT3-U6- sgGabrd-EF1a-Cas9, one empty vector control group received pT3-EF1a-NC, and the other control group received pT3-U6-sgNC-EF1a-Cas9. The total volume of the plasmid mixture per mouse was adjusted to 8–10% of the mouse’s body weight in sterile saline solution, and the injection was completed within 5–10 s through the tail vein to maintain hydrodynamic pressure. The Sleeping Beauty transposon system enables stable genomic integration and sustained expression of the transgenes. At 4 weeks post-injection, the mice were sacrificed by cervical dislocation. Livers were immediately dissected to count the number of tumor nodules per mouse. The length and width of each nodule were measured using a vernier caliper, and tumor volume was calculated using the formula V = (length×width^2^)/2. Tumor tissues were harvested for IHC to validate the genetic manipulations.

### Lung metastasis model

C57BL/6 mice (6–8 weeks old) were randomly assigned to groups (*n* = 5 per group). To establish lung metastases, 1 × 10⁶ Hepa1-6 cells with stable GABRD knockout (Hepa1-6-GABRD-KO) or overexpression (Hepa1-6-GABRD-OE) were suspended in 100 µL of PBS and injected into the tail vein. Six weeks later, lung metastasis was monitored and quantified using an in vivo bioluminescence imaging (IVIS) system after intraperitoneal injection of D-luciferin (150 mg/kg). Subsequently, the mice were euthanized, and their lungs were excised, fixed in 10% formalin, and embedded in paraffin. Metastatic foci were confirmed and quantified on hematoxylin and eosin (HE)-stained tissue sections.

### Pharmacological intervention

The JAK2-STAT3 inhibitor WP1066 was used to treat GABRD-overexpressing HuH-7 cells in vitro and tumor-bearing mice in vivo; for in vitro experiments, cells were treated with 10 μM WP1066 dissolved in DMSO or vehicle control (DMSO at a final concentration of 0.1%) for 48 h, and cell proliferation, invasion, and migration were assessed, while for in vivo experiments, WP1066 was dissolved in DMSO and diluted in sterile saline containing 10% 2-hydroxypropyl-β-cyclodextrin, and tumor-bearing mice received intraperitoneal injections of WP1066 at 40 mg/kg or vehicle control every other day for 4 weeks starting one week after tumor cell inoculation, with tumor growth being monitored. Additionally, in IL-10RA neutralization experiments, mice bearing Gabrd-OE tumors in the Myc/AKT HCC model were intraperitoneally injected with anti-IL-10RA neutralizing antibody (10 mg/kg) or sterile saline twice weekly for 4 weeks, beginning one week after hydrodynamic transfection.

### Co-immunoprecipitation (Co-IP)

HuH-7 cells with stable GABRD overexpression were cultured to 80–90% confluence. Wash with PBS, lyse with RIPA buffer containing protease, and centrifuge to collect the supernatant. Dilute 500 μg of protein with IP buffer, pre-clear with Protein A/G agarose beads, and then incubate with anti-GABRD antibody or normal rabbit IgG overnight. The next day, add more beads to form complexes, wash five times to remove non-specific proteins, and elute the immunoprecipitated proteins with 2× SDS loading buffer by boiling. Analyze the eluted samples by SDS-PAGE and Western blot to verify the interaction between GABRD and other candidate proteins.

### Mass spectrometry analysis

Separate Co-IP complexes by SDS-PAGE, stain and destain the gel, excise target bands, and process the gel pieces for in-gel digestion with trypsin. Collect and dry the resulting peptides, reconstitute them, and analyze them by nano-LC-MS/MS. Search the MS/MS data against the UniProt human protein database using MaxQuant, identifying proteins with FDR < 1%. Screen GABRD-interacting proteins based on presence in antibody samples but not controls, at least 2 unique peptides, and PSMs ≥3.

### Molecular docking analysis

Rigid protein–protein docking was performed via AlphaFold3, with fixed 3D conformations of receptor and ligand proteins, and the algorithm searched for the optimal geometric binding interface on the receptor surface; for the detailed experimental procedure, the full-length amino acid sequences of target proteins GABRD (UniProt ID: O14764) and IL‑10 RA (UniProt ID: Q13651) were first retrieved from UniProtKB (https://www.uniprot.org/) by gene symbols, then the sequences of the two proteins were input into AlphaFold3, docking was conducted with default protein–protein parameters, and the top five docking models (ranked by binding quality) were collected with rank 1 as the optimal conformation, finally, binding free energy was analyzed combined with interaction surface area, hydrogen bonds and key amino acid residues, and the binding energy was calculated by PDBePISA while visualization was performed by PyMOL 3.1.

### Statistical analysis

All experiments were performed at least three times independently. Data were presented as mean ± standard deviation (SD). Statistical analysis was performed using SPSS 26.0 (IBM Corp., Armonk, NY, USA) and GraphPad Prism 9.0 (GraphPad Software, San Diego, CA, USA). For comparisons between two groups, the unpaired two-tailed Student’s *t*-test was used after confirming normality by the Shapiro–Wilk test and homogeneity of variance by Levene’s test. For multiple group comparisons, one‑way analysis of variance (ANOVA) followed by Tukey’s post hoc test was applied. For all analyses, a value of *p* < 0.05 was considered statistically significant, and all tests were two‑sided. Sample sizes were determined based on power analysis (power = 0.8, α = 0.05) using G*Power 3.1.

## Discussion

Our findings reveal that GABRD as a critical promoter of HCC progression. We demonstrate that GABRD is markedly upregulated in HCC tissues, where its high expression correlates with poor patient prognosis. Mechanistically, GABRD drives malignant phenotypes by forming a functional complex with IL-10RA, thereby activating the JAK2-STAT3 signaling cascade and inducing EMT. These findings uncover a novel mechanism in HCC and highlight the GABRD-IL-10RA-JAK2-STAT3 axis as a promising target for therapeutic intervention.

Neurotransmitter receptors, traditionally studied in the context of neuronal signaling, have increasingly been recognized as critical modulators of tumor progression [31]. Glutamate receptors, particularly NMDA and AMPA subtypes, are reported to drive tumor growth, and metabolic reprogramming through activation of MAPK, and calcium-dependent pathways [32–34]. Cholinergic signaling via nicotinic and muscarinic acetylcholine receptors can enhance proliferation, angiogenesis, and metastasis in various solid tumors, with nicotine-mediated receptor activation particularly relevant in smoking-related cancers [35, 36]. Among these, γ-aminobutyric acid (GABA) receptors have been extensively investigated for their dualistic roles in cancer biology. GABA regulates microenvironmental homeostasis predominantly via ionotropic GABA_A_ receptors and metabotropic GABA_B_ receptor signaling [10, 37]. GABRD, a critical functional subunit of the GABA_A receptor, is frequently aberrantly expressed in a broad range of human malignancies. Accumulating evidence has demonstrated that GABRD facilitates the malignant progression of gastric cancer and colon adenocarcinoma, highlighting a neurotransmitter-dependent regulatory mechanism underlying tumorigenesis and tumor progression [17, 21]. Moreover, GPT2, a key rate-limiting enzyme for GABA biosynthesis, promotes autocrine GABA production, which further activates membrane-expressed GABRD to drive breast cancer metastasis [18].

In addition to canonical GABA-secreting neuronal cells, emerging studies have verified that multiple immune cell subsets within the tumor immune microenvironment, such as B cells [38], are capable of producing and secreting GABA to activate downstream GABA receptor-related signaling cascades. In the present study, we preliminarily clarified the oncogenic role of GABRD in facilitating HCC progression. Nevertheless, it remains poorly defined whether other stromal or immune cell populations in the HCC microenvironment can secrete GABA to trigger the GABRD/IL-10RA-JAK-STAT signaling axis. Accordingly, this scientific question will be systematically explored in our future investigations.

In the present study, we demonstrated that the GABRD/IL-10RA complex facilitates HCC proliferation, invasion and metastasis by activating the downstream JAK2-STAT3 signaling axis. It is well established that the functional activation of IL-10R is strongly dependent on stimulation by microenvironmental IL-10. Accordingly, our findings indicate that the tumor immune microenvironment is critically involved in the oncogenic effects mediated by the GABRD/IL-10RA axis.

As a pivotal immunosuppressive cytokine, IL-10 is documented to be secreted by multiple distinct immune cell populations within the tumor microenvironment. Tumor-associated macrophage-derived IL-10 acts on tumor-associated neutrophils to establish an immunosuppressive microenvironment and thereby accelerate tumor progression [39]. Foxp3⁺ regulatory T cell (Treg)-derived IL-10 restrains the antitumor activity of CD8⁺ T cells by upregulating PD-L1, consequently promoting colorectal cancer liver metastasis [40]. In diffuse large B-cell lymphoma, tumoral B cell-secreted IL-10 remodels an immunosuppressive microenvironment via STAT3 activation, impairs effector T cell function, drives lymphoma progression, and induces immunotherapy resistance [41]. In addition, activation of GABA receptors has been shown to promote the differentiation of monocytes into anti-inflammatory, IL-10-producing macrophages, while suppressing the cytotoxic capacity of CD8⁺ T cells to exert broad immunosuppressive effects [42]. Nevertheless, the cellular origin of microenvironmental IL-10 and the detailed cell–cell interaction patterns were not investigated in the current study. Therefore, the specific molecular mechanisms by which the IL-10–IL-10RA/GABRD cascade mediates tumor immune suppression will be further elucidated in our future research.

IL-10R signaling is typically initiated by microenvironmental IL-10 ligation, which subsequently triggers downstream pro-tumorigenic cascades. Accumulating clinical evidence has revealed that elevated IL-10R expression is closely correlated with poor patient prognosis, and targeted inhibition of IL-10R effectively restrains tumor progression [43, 44]. Hence, alterations in IL-10R expression levels alone are sufficient to modulate HCC malignant progression. Although the present study did not further explore the cell–cell interaction mechanism by which HCC membrane-localized IL-10R is activated by upstream IL-10 in the tumor microenvironment, such a deficiency does not compromise the validity and reliability of our current conclusions.

We have provided compelling evidence for a direct physical interaction between GABRD and IL-10RA. We further revealed a unique unidirectional hierarchical regulatory pattern: genetic ablation of GABRD led to a significant reduction in IL-10RA protein levels without affecting its mRNA abundance, whereas the loss of IL-10RA had no impact on GABRD expression. These observations suggest that GABRD may regulate IL-10RA protein stability through post-translational mechanisms. The ubiquitin-proteasome system, as well as acetylation and succinylation modifications that coordinate with ubiquitination, together with the autophagy-lysosome pathway, are all critical participants in the post-translational regulation of protein homeostasis [45–47]. Whether GABRD mediates the expression of IL-10RA at the protein level through the above-mentioned pathways, either directly or indirectly, remains to be clarified. In addition, alterations in IL-10R expression can profoundly affect its homodimerization or heteromultimerization, thereby governing the activation of the IL-10–IL-10R signaling cascade and downstream biological responses.

Notably, whether GABRD restructures IL-10R multimerization via post-translational modifications to trigger immunosuppression within the tumor immune microenvironment and facilitate HCC malignant progression still requires in-depth exploration. Therefore, our future work will further elucidate the oncogenic mechanisms of the GABRD/IL-10RA axis by focusing on tumor microenvironment remodeling and post-translational modification regulation.

The clinical significance of our findings necessitates further investigation with expanded patient cohorts to determine the correlation between GABRD and IL-10R expression in HCC. Our analysis of TCGA-LIHC data and a limited patient cohort demonstrated that GABRD upregulation correlates with poor prognosis, consistent with observations in colorectal cancer, where GABRD expression is associated with adverse clinical outcomes and immune phenotypes. Similar analyses in HCC should evaluate whether GABRD and IL-10RA expression levels correlate with each other and with key clinicopathological parameters, such as tumor grade, vascular invasion, and recurrence. Furthermore, investigating their co-expression patterns within the TME could yield valuable prognostic insights. Integrating these clinical correlations with our functional data could identify a subset of HCC patients who would benefit from targeted therapies against the GABRD/IL-10RA axis.

However, a key limitation of the present study is that we primarily focused on tumor cell-intrinsic mechanisms of the GABRD/IL-10RA axis in HCC, without extending our investigations to its specific effects on tumor-infiltrating immune cells, such as macrophages and T cells. Furthermore, the specific post-translational modification patterns through which GABRD regulates IL-10RA protein levels remain unaddressed. These knowledge gaps hinder a comprehensive understanding of the GABRD/IL-10RA axis in shaping the tumor immune microenvironment. Therefore, we will conduct an in-depth exploration of these issues in our follow-up research.

In summary, our study identifies the GABRD-IL-10RA-JAK2-STAT3 axis as a novel and critical driver of HCC progression. We demonstrate that GABRD acts as a key oncogenic hub, and its inhibition or the disruption of its interaction with IL-10RA effectively suppresses tumor growth. These findings position GABRD as a promising therapeutic target. Moreover, our data suggest that a combined therapeutic strategy, pairing GABRD-targeted agents with JAK2-STAT3 inhibitors, holds significant potential to overcome therapeutic resistance and improve outcomes for patients with HCC.

## Supplementary information

Original Western blots

GABRD promotes hepatocellular carcinoma progression via the IL-10RA/JAK2-STAT3 signaling axis

## Data Availability

The data that support the findings of this study are available from the corresponding author upon reasonable request.

## References

[1] Bray F, Laversanne M, Sung H, Ferlay J, Siegel RL, Soerjomataram I, et al. Global cancer statistics 2022: GLOBOCAN estimates of incidence and mortality worldwide for 36 cancers in 185 countries. CA Cancer J Clin. 2024;74:229–63. 38572751 10.3322/caac.21834

[2] He T, Zou J, Sun K, Yang J. Global research status and frontiers on autophagy in hepatocellular carcinoma: a comprehensive bibliometric and visualized analysis. Int J Surg. 2024;110:2788–802. 38376850 10.1097/JS9.0000000000001202 PMC11093451

[3] McGlynn KA, Petrick JL, El-Serag HB. Epidemiology of hepatocellular carcinoma. Hepatology. 2021;73:4–13. 32319693 10.1002/hep.31288 PMC7577946

[4] Sun Y, Wang X, Zhang DY, Zhang Z, Bhattarai JP, Wang Y, et al. Brain-wide neuronal circuit connectome of human glioblastoma. Nature. 2025;641:222–31. 39821165 10.1038/s41586-025-08634-7 PMC12347542

[5] Liu K, Zhang Y, Du G, Chen X, Xiao L, Jiang L, et al. 5-HT orchestrates histone serotonylation and citrullination to drive neutrophil extracellular traps and liver metastasis. J Clin Invest. 2025;135:e183544. 39903533 10.1172/JCI183544 PMC11996869

[6] Huang Q, Hu B, Zhang P, Yuan Y, Yue S, Chen X, et al. Neuroscience of cancer: unraveling the complex interplay between the nervous system, the tumor and the tumor immune microenvironment. Mol Cancer. 2025;24:24. 39825376 10.1186/s12943-024-02219-0 PMC11740516

[7] Bucsek MJ, Qiao G, MacDonald CR, Giridharan T, Evans L, Niedzwecki B, et al. β-adrenergic signaling in mice housed at standard temperatures suppresses an effector phenotype in CD8(+) T cells and undermines checkpoint inhibitor therapy. Cancer Res. 2017;77:5639–51. 28819022 10.1158/0008-5472.CAN-17-0546 PMC5645237

[8] Kuol N, Davidson M, Karakkat J, Filippone RT, Veale M, Luwor R, et al. Blocking muscarinic receptor 3 attenuates tumor growth and decreases immunosuppressive and cholinergic markers in an orthotopic mouse model of colorectal cancer. Int J Mol Sci. 2022;24:596. 36614038 10.3390/ijms24010596 PMC9820315

[9] Dong Y, Wang G, Nie D, Xu Y, Bai X, Lu C, et al. Tumor-derived GABA promotes lung cancer progression by influencing TAMs polarization and neovascularization. Int Immunopharmacol. 2024;126:111217. 37977069 10.1016/j.intimp.2023.111217

[10] Qian X, Zhao X, Yu L, Yin Y, Zhang XD, Wang L, et al. Current status of GABA receptor subtypes in analgesia. Biomed Pharmacother. 2023;168:115800. 37935070 10.1016/j.biopha.2023.115800

[11] Ahring PK, Liao VWY, Gardella E, Johannesen KM, Krey I, Selmer KK, et al. Gain-of-function variants in GABRD reveal a novel pathway for neurodevelopmental disorders and epilepsy. Brain. 2022;145:1299–309. 34633442 10.1093/brain/awab391 PMC9630717

[12] Granados-Fuentes D, Lambert P, Simon T, Mennerick S, Herzog ED. GABA(A) receptor subunit composition regulates circadian rhythms in rest-wake and synchrony among cells in the suprachiasmatic nucleus. Proc Natl Acad Sci USA. 2024;121:e2400339121. 39047036 10.1073/pnas.2400339121 PMC11295074

[13] Guo Z, Tian C, Shi Y, Song XR, Yin W, Tao QQ, et al. Blood-based CNS regionally and neuronally enriched extracellular vesicles carrying pTau217 for Alzheimer’s disease diagnosis and differential diagnosis. Acta Neuropathol Commun. 2024;12:38. 38444036 10.1186/s40478-024-01727-w PMC10913681

[14] Luscher B, Maguire JL, Rudolph U, Sibille E. GABA(A) receptors as targets for treating affective and cognitive symptoms of depression. Trends Pharmacol Sci. 2023;44:586–600. 37543478 10.1016/j.tips.2023.06.009 PMC10511219

[15] Lin BB, Huang Q, Yan B, Liu M, Zhang Z, Lei H, et al. An 18-gene signature of recurrence-associated endothelial cells predicts tumor progression and castration resistance in prostate cancer. Br J Cancer. 2024;131:870–82. 38997406 10.1038/s41416-024-02761-0 PMC11369112

[16] Yuan Y, Ping W, Zhang R, Hao Z, Zhang N. DEPDC1B collaborates with GABRD to regulate ESCC progression. Cancer Cell Int. 2022;22:214. 35706026 10.1186/s12935-022-02593-z PMC9202211

[17] Leng W, Ye J, Wen Z, Wang H, Zhu Z, Song X, et al. GABRD accelerates tumour progression via regulating CCND1 signalling pathway in gastric cancer. J Cell Mol Med. 2025;29:e70485. 40145254 10.1111/jcmm.70485 PMC11947670

[18] Li N, Xu X, Liu D, Gao J, Gao Y, Wu X, et al. The delta subunit of the GABA(A) receptor is necessary for the GPT2-promoted breast cancer metastasis. Theranostics. 2023;13:1355–69. 36923530 10.7150/thno.80544 PMC10008743

[19] Wu M, Kim KY, Park WC, Ryu HS, Choi SC, Kim MS, et al. Enhanced expression of GABRD predicts poor prognosis in patients with colon adenocarcinoma. Transl Oncol. 2020;13:100861. 32891902 10.1016/j.tranon.2020.100861 PMC7484591

[20] Zhang L, Deng Y, Yang J, Deng W, Li L. Neurotransmitter receptor-related gene signature as potential prognostic and therapeutic biomarkers in colorectal cancer. Front Cell Dev Biol. 2023;11:1202193. 38099288 10.3389/fcell.2023.1202193 PMC10720326

[21] Huang F, Wang Z, Zhu L, Lin C, Wang JX. Comprehensive analysis of the expression, prognostic value, and immune infiltration activities of GABRD in colon adenocarcinoma. Mediators Inflamm. 2023;2023:8709458. 37181811 10.1155/2023/8709458 PMC10169248

[22] Liu RY, Zeng Y, Lei Z, Wang L, Yang H, Liu Z, et al. JAK/STAT3 signaling is required for TGF-β-induced epithelial-mesenchymal transition in lung cancer cells. Int J Oncol. 2014;44:1643–51. 24573038 10.3892/ijo.2014.2310

[23] Shih PC, Mei KC. Role of STAT3 signaling transduction pathways in cancer stem cell-associated chemoresistance. Drug Discov Today. 2021;26:1450–8. 33307211 10.1016/j.drudis.2020.11.032

[24] Fan AC, Nakauchi Y, Bai L, Azizi A, Nuno KA, Zhao F, et al. RUNX1 loss renders hematopoietic and leukemic cells dependent on IL-3 and sensitive to JAK inhibition. J Clin Invest. 2023;133:e167053. 37581927 10.1172/JCI167053 PMC10541186

[25] Holzgruber J, Martins C, Kulcsar Z, Duplaine A, Rasbach E, Migayron L, et al. Type I interferon signaling induces melanoma cell-intrinsic PD-1 and its inhibition antagonizes immune checkpoint blockade. Nat Commun. 2024;15:7165. 39187481 10.1038/s41467-024-51496-2 PMC11347607

[26] Wang X, Wong K, Ouyang W, Rutz S. Targeting IL-10 family cytokines for the treatment of human diseases. Cold Spring Harb Perspect Biol. 2019;11:a028548. 29038121 10.1101/cshperspect.a028548 PMC6360861

[27] Sun Z, Xu Y, Shao B, Dang P, Hu S, Sun H, et al. Exosomal circPOLQ promotes macrophage M2 polarization via activating IL-10/STAT3 axis in a colorectal cancer model. J Immunother Cancer. 2024;12:e008491. 38782541 10.1136/jitc-2023-008491 PMC11116870

[28] Chen X, Calvisi DF. Hydrodynamic transfection for generation of novel mouse models for liver cancer research. Am J Pathol. 2014;184:912–23. 24480331 10.1016/j.ajpath.2013.12.002 PMC3969989

[29] Sun Y, Xu M, Wan HL, Ding X, Wong AM, Pu D, et al. Spliced exon9 ADRM1 promotes liver oncogenicity via selective degradation of tumor suppressor FBXW7. J Hepatol. 2025;83:92–104. 39788431 10.1016/j.jhep.2024.12.037

[30] Zheng C, Snow BE, Elia AJ, Nechanitzky R, Dominguez-Brauer C, Liu S, et al. Tumor-specific cholinergic CD4(+) T lymphocytes guide immunosurveillance of hepatocellular carcinoma. Nat Cancer. 2023;4:1437–54. 37640929 10.1038/s43018-023-00624-w PMC10597839

[31] Anastasaki C, Mu R, Kernan CM, Li X, Barakat R, Koleske JP, et al. Aberrant coupling of glutamate and tyrosine kinase receptors enables neuronal control of brain-tumor growth. Neuron. 2025;113:3582–600.e7. 40897174 10.1016/j.neuron.2025.08.005 PMC12416319

[32] Dai WL, Bao YN, Fan JF, Ma B, Li SS, Zhao WL, et al. Blockade of spinal dopamine D1/D2 receptor suppresses activation of NMDA receptor through Gαq and Src kinase to attenuate chronic bone cancer pain. J Adv Res. 2021;28:139–48. 33364051 10.1016/j.jare.2020.08.005 PMC7753228

[33] Zhang Y, Liu D, Guo D, Lin W, Lu W, Hu L, et al. CPSF3 regulates alternative polyadenylation of CNIH2 to promote esophageal squamous cell carcinoma progression. Cancer Lett. 2024;593:216925. 38718887 10.1016/j.canlet.2024.216925

[34] Ren L, Liu C, Çifcibaşı K, Ballmann M, Rammes G, Mota Reyes C, et al. Sensory neurons drive pancreatic cancer progression through glutamatergic neuron-cancer pseudo-synapses. Cancer Cell. 2025;43:2241–58.e8. 41005304 10.1016/j.ccell.2025.09.003

[35] Kasheverov IE, Shelukhina IV, Utkin YN, Tsetlin VI. Marine-derived ligands of nicotinic acetylcholine receptors in cancer research. Mar Drugs. 2025;23:389. 41149592 10.3390/md23100389 PMC12565509

[36] Zhang C, Guo X, Wang Y, Zhang S, Wang Z. The role of acetylcholine and its receptors in tumor immune regulation: mechanisms and potential therapeutic targets. Mol Cancer. 2025;24:231. 41029375 10.1186/s12943-025-02441-4 PMC12482714

[37] Huang D, Wang Y, Thompson JW, Yin T, Alexander PB, Qin D, et al. Cancer-cell-derived GABA promotes β-catenin-mediated tumour growth and immunosuppression. Nat Cell Biol. 2022;24:230–41. 35145222 10.1038/s41556-021-00820-9 PMC8852304

[38] Zhang B, Vogelzang A, Miyajima M, Sugiura Y, Wu Y, Chamoto K, et al. B cell-derived GABA elicits IL-10(+) macrophages to limit anti-tumour immunity. Nature. 2021;599:471–6. 34732892 10.1038/s41586-021-04082-1 PMC8599023

[39] Chang Z, Guo X, Li X, Wang Y, Zang Z, Pei S, et al. Bacterial immunotherapy leveraging IL-10R hysteresis for both phagocytosis evasion and tumor immunity revitalization. Cell. 2025;188:1842–57.e20. 40037354 10.1016/j.cell.2025.02.002

[40] Shiri AM, Zhang T, Bedke T, Zazara DE, Zhao L, Lücke J, et al. IL-10 dampens antitumor immunity and promotes liver metastasis via PD-L1 induction. J Hepatol. 2024;80:634–44. 38160941 10.1016/j.jhep.2023.12.015 PMC10964083

[41] Garcia-Lacarte M, Grijalba SC, Melchor J, Pascual M, Goñi E, Clemente-Larramendi I, et al. IL-10 from tumoral B cells modulates the diffuse large B-cell lymphoma microenvironment and response to immunotherapy. Blood. 2025;145:2746–61. 39899878 10.1182/blood.2024025755 PMC12824703

[42] Jiang SH, Zhu LL, Zhang M, Li RK, Yang Q, Yan JY, et al. GABRP regulates chemokine signalling, macrophage recruitment and tumour progression in pancreatic cancer through tuning KCNN4-mediated Ca(2+) signalling in a GABA-independent manner. Gut. 2019;68:1994–2006. 30826748 10.1136/gutjnl-2018-317479

[43] Du W, Ouyang Y, Feng X, Yu C, Zhang H, Chen S, et al. IL-10RA promotes lung cancer cell proliferation by increasing fatty acid oxidation via STAT3 signaling pathway. Pulm Pharmacol Ther. 2025;88:102344. 39892560 10.1016/j.pupt.2025.102344

[44] Sun J, Corradini S, Azab F, Shokeen M, Muz B, Miari KE, et al. IL-10R inhibition reprograms tumor-associated macrophages and reverses drug resistance in multiple myeloma. Leukemia. 2024;38:2355–65. 39215060 10.1038/s41375-024-02391-8 PMC11518999

[45] Shu F, Xiao H, Li QN, Ren XS, Liu ZG, Hu BW, et al. Epigenetic and post-translational modifications in autophagy: biological functions and therapeutic targets. Signal Transduct Target Ther. 2023;8:32. 36646695 10.1038/s41392-022-01300-8 PMC9842768

[46] Yang N, Li L, Shi XL, Liu YP, Wen R, Yang YH, et al. Succinylation of SERCA2a at K352 promotes its ubiquitinoylation and degradation by proteasomes in sepsis-induced heart dysfunction. Circ Heart Fail. 2025;18:e012180. 39996319 10.1161/CIRCHEARTFAILURE.124.012180

[47] Lee JM, Hammarén HM, Savitski MM, Baek SH. Control of protein stability by post-translational modifications. Nat Commun. 2023;14:201. 36639369 10.1038/s41467-023-35795-8 PMC9839724

