# Supplementary material for: GABRD promotes hepatocellular carcinoma progression via the IL-10RA/JAK2-STAT3 signaling axis
Source: Cell Death Dis. 2026 Jun 8;17(1):720. doi: 10.1038/s41419-026-08969-7 (PMC13470470; doi:10.1038/s41419-026-08969-7)
Supplement: Supplementary file 1 [file 41419_2026_8969_MOESM1_ESM.docx]

**Figure 1.**

**H. GABRD in HCC tissues**

25kDa

33kDa

43kDa

25kDa

33kDa

43kDa

130kDa

95kDa

55kDa

70kDa

**I. GABRD in HCC cell lines and L68**

33kDa

43kDa

55kDa

70kDa

**Figure 5.**

**D. EMT markers of NTC vs. GABRD-KO**

70kDa

55kDa

130kDa

180kDa

55kDa

70kDa

180kDa

130kDa

33kDa

**E. EMT markers of control vs. GABRD**

55kDa

70kDa

130kDa

180kDa

55kDa

33kDa

33kDa

180kDa

130kDa

**J. JAK2-STAT3 of control vs. GABRD**

95kDa

130kDa

70kDa

95kDa

130kDa

180kDa

43kDa

55kDa

33kDa

130kDa

180kDa

25kDa

33kDa

55kDa

70kDa

95kDa

70kDa

55kDa

**K. JAK2-STAT3 of NTC vs. GABRD-KO**

33kDa

25kDa

43kDa

130kDa

180kDa

95kDa

55kDa

70kDa

95kDa

95kDa

180kDa

130kDa

55kDa

70kDa

95kDa

55kDa

70kDa

95kDa

55kDa

70kDa

95kDa

43kDa

25kDa

33kDa

**Figure 6.**

**A. JAK2-STAT3 of OE, OE+DMSO and OE+WP1066**

55kDa

70kDa

95kDa

95kDa

70kDa

130kDa

180kDa

25kDa

33kDa

43kDa

95kDa

130kDa

180kDa

70kDa

43kDa

33kDa

25kDa

95kDa

130kDa

180kDa

**Figure 7.**

**C. CO-IP of candidate proteins**

130kDa

95kDa

55kDa

43kDa

33kDa

43kDa

55kDa

43kDa

55kDa

70kDa

33kDa

43kDa

55kDa

70kDa

55kDa

**D. Reciprocal CO-IP of GABRD and IL-10RA**

55kDa

70kDa

95kDa

130kDa

43kDa

33kDa

33kDa

43kDa

55kDa

70kDa

95kDa

**F. JAK2, STAT3 and EMT markers in Control, GABRD-OE and GABRD-OE+IL-10KO**

70kDa

55kDa

95kDa

95kDa

130kDa

180kDa

55kDa

70kDa

95kDa

55kDa

95kDa

70kDa

33kDa

43kDa

95kDa

180kDa

130kDa

55kDa

70kDa

55kDa

70kDa

95kDa

95kDa

180kDa

130kDa

**Figure S2.**

**Establishment of cell lines**

55kDa

70kDa

95kDa

95kDa

55kDa

70kDa

95kDa

55kDa

70kDa

95kDa

55kDa

70kDa

43kDa

33kDa

33kDa

43kDa

33kDa

43kDa

33kDa

43kDa

**Figure S5.**

**phosphorylated JAK1, STAT1 and STAT5**

95kDa

70kDa

55kDa

43kDa

33kDa

70kDa

95kDa

95kDa

95kDa

70kDa

95kDa

130kDa

95kDa

130kDa

180kDa

70kDa

55kDa

55kDa

70kDa

**Figure S6.**

**JAK2, STAT3, E-cadherin，Vimentin, and GABRD**

55kDa

70kDa

95kDa

43kDa

33kDa

25kDa

130kDa

95kDa

180kDa

55kDa

43kDa

70kDa

95kDa

95kDa

130kDa

180kDa

95kDa

55kDa

70kDa

70kDa

55kDa

130kDa

180kDa

95kDa

**Figure S7.**

**A. JAK2, STAT3, IL-10RA, and GABRD**

95kDa

70kDa

55kDa

70kDa

95kDa

33kDa

25kDa

43kDa

70kDa

55kDa

95kDa

43kDa

55kDa

70kDa

95kDa

130kDa

95kDa

180kDa

95kDa

130kDa

180kDa

**B. IL-10RA, and GABRD in GABRD-KO**

33kDa

43kDa

55kDa

43kDa

70kDa

95kDa

55kDa

70kDa

95kDa

130kDa

**C. IL-10RA, and GABRD in IL-10RA-KO**

33kDa

43kDa

130kDa

70kDa

95kDa

55kDa

43kDa

55kDa

70kDa
