# Supplementary material for: GABRD promotes hepatocellular carcinoma progression via the IL-10RA/JAK2-STAT3 signaling axis
Source: Cell Death Dis. 2026 Jun 8;17(1):720. doi: 10.1038/s41419-026-08969-7 (PMC13470470; doi:10.1038/s41419-026-08969-7)
Supplement: Supplementary file 2 [file 41419_2026_8969_MOESM2_ESM.docx]

## GABRD promotes hepatocellular carcinoma progression via the IL-10RA/JAK2-STAT3 signaling axis

Chengcheng Huang^1,†^, Weiwei Liu^1,†^, Zheng Wang^1,†^, Yingling Liu^2^, Zhenfei Bi^1^, Bin Han^1^, Yue Yan^1^, Shuqi Zhang^1^, Boyu Zhu^1^, Ziyi Zhou^1^, Chaoqun Wang^1,*^, Lu Zheng^1,*^, Jing Li^1,*^

^1^Key Laboratory for Targeted Therapy of Hepatopancreatobiliary Diseases of Chongqing, Department of Hepatopancreatobiliary Surgery, The Second Affiliated Hospital of Army Medical University, Chongqing 400037, China;

^2^Department of General Surgery, Xinhua Hospital, Affiliated to Shanghai Jiao Tong University School of Medicine, Shanghai, 200092, China

**Corresponding Authors:** Li Jing, E-mail: [xqyylj@tmmu.edu.cn](mailto:xqyylj@tmmu.edu.cn) ; Lu Zheng, E-mail: [zhenglu@tmmu.edu.cn](mailto:zhenglu@tmmu.edu.cn) ; Wang Chaoqun, E-mail: [wangchq92@tmmu.edu.cn](mailto:wangchq92@tmmu.edu.cn), Key Laboratory for Targeted Therapy of Hepatopancreatobiliary Diseases of Chongqing, Department of Hepatopancreatobiliary Surgery, The Second Affiliated Hospital of Army Medical University, Chongqing 400037, China

**Supplementary table 1. Primers used for qPCR**

| **Gene** | **Primer Direction** | **Sequence (5’-3’)** |
| --- | --- | --- |
| GABRD | Forward | CAGCATCGACCACATCTCAG |
| GABRD | Reverse | TCGTTGGTGTGGTTGTAGGA |
| GAPDH | Forward | TCCTCCGGGTGATGCTTTTC |
| GAPDH | Reverse | GCAGGAGGCATTGCTGATGA |

**Supplementary table 2. Primary antibodies**

| **Target** | **Catalog Number** | | **Company** | | **Application** |
| --- | --- | --- | --- | --- | --- |
| GABRD | | sc-271231 | | Santa Cruz Biotechnology | WB, IF |
| GAPDH | | 10494-1-AP | | Proteintech | WB, IF |
| JAK2 | | 3230 | | Cell Signaling Technology | WB, IF |
| Phospho-JAK2 (Tyr1007/1008) | | 3771 | | Cell Signaling Technology | WB, IF |
| STAT3 | | 12640 | | Cell Signaling Technology | WB, IF |
| Phospho-STAT3 (Tyr705) | | 9145 | | Cell Signaling Technology | WB, IF |
| E-cadherin | | 20874-1-AP | | Proteintech | WB, IF |
| N-cadherin | | 22018-1-AP | | Proteintech | WB, IF |
| Vimentin | | 60330-1-Ig | | Proteintech | WB, IF |
| IL-10RA | | 13356-1-AP | | Proteintech | WB, IF |
| TUBB2A | | sc-47751 | | Santa Cruz Biotechnology | WB, IF |
| COPE | | 11457-1-AP | | Proteintech | WB, IF |
| TMCO1 | | 27757-1-AP | | Proteintech | WB, IF |
| SLC2A2 | | 20436-1-AP | | Proteintech | WB, IF |
| NRP1 | | 60067-1-Ig | | Proteintech | WB, IF |
| Ki67 | | ab16667 | | ABclonal | IHC |

**Supplementary table 3. Other reagents**

| **Reagent** | **Catalog Number** | **Company** | **Application / Treatment** |
| --- | --- | --- | --- |
| JAK2/STAT3 inhibitor (WP1066) | HY-15312 | MCE | Dissolved in DMSO as stock; diluted in culture medium for in vitro use. |
| Anti-mouse IL-10RA neutralizing antibody | BE0050 | Bio Cell | 40 mg/kg, intraperitoneal injection, twice weekly (in vivo). |

**Supplementary table 4. sgRNA sequences for GABRD knockout**

| **Species** | **Gene** | **Targeted Exon** | **sgRNA Sequence (5’ -> 3’)** | **PAM** |
| --- | --- | --- | --- | --- |
| Human | SgGABRD1 | Exon 2 | GGCTCAGCTGATGGATCTGG | AGG |
| Human | SgGABRD2 | Exon 3 | CAGTTCATCTTCGAGAACCT | TGG |
| Mouse | SgGabrd1 | Exon 3 | GGAGCTGCTGAGGAGGCTCA | GGG |
| Mouse | SgGabrd2 | Exon 1 | GCTACGAGATCGACTACGAG | AGG |

**Supplementary table 5. sgRNA sequences for IL-10RA knockout**

| **Species** | **Gene** | **Targeted Exon** | **sgRNA Sequence (5’ -> 3’)** | **PAM** |
| --- | --- | --- | --- | --- |
| Human | sgIL-10RA1 | Exon 2 | GATGACAAGTTCCAGAACCG | TGG |
| Human | sgIL-10RA2 | Exon 3 | GGATTCTTCTGAAGAGCTGT | TGG |

**Supplementary table 6. Candidate proteins interacting with GABRD**

| **Gene names** | **Protein score** | **Coverage [%]** | **Peptides** | **PSMs** | **Unique Peptides** | **MW [kDa]** |
| --- | --- | --- | --- | --- | --- | --- |
| TUBB2A | 71.008 | 32 | 12 | 52 | 1 | 49.9 |
| IL10RA | 36.249 | 15 | 8 | 18 | 8 | 70 |
| COPE | 24.094 | 12 | 3 | 7 | 3 | 34.5 |
| TMCO1 | 6.955 | 6 | 1 | 3 | 1 | 27.1 |
| SLC2A2 | 5.762 | 4 | 2 | 2 | 2 | 57.5 |
| NRP1 | 5.321 | 3 | 2 | 2 | 2 | 103.1 |

**Supplementary figure 1**

**Supplementary figure 1. GABRD is upregulated in HCC and correlates with poor prognosis**

**A.** External validation of Overall survival in HCC patients with high/low GABRD expression in GEO dataset.

**Supplementary figure 2**

**Supplementary figure 2. Establishment of stable GABRD cell lines**

**A, B.** Immunoblotting (**A**) and qPCR analysis (**B**) of GABRD protein and mRNA expression in HCCLM3-GABRD-KO vs. HCCLM3-NTC. **C, D.** Immunoblotting (**C**) and qPCR analysis (**D**) of GABRD protein and mRNA expression in MHCC97H-GABRD-KO vs. MHCC97H-NTC. **E, F.** Immunoblotting (**E**) and qPCR analysis (**F**) of GABRD protein and mRNA expression in HuH-7-GABRD vs. HuH-7-control. **G, H.** Immunoblotting (**G**) and qPCR analysis (**H**) of GABRD protein and mRNA expression in SUN-449-GABRD vs. SUN-449-control. Statistical significance was determined by two-tailed Student's t-test, n=3. *P < 0. 05, **P < 0. 01, ***P < 0. 001 ****P < 0. 0001 and ns, no significance.

**Supplementary figure 3**

**Supplementary figure 3. GABRD promotes HCC proliferation in *vivo***

**A, B** Immunohistochemical staining and mean optical density (MOD) quantification of GABRD in subcutaneous tumor tissues from GABRD-KO vs. NTC (**A**) and GABRD-OE vs. Control (**B**) groups, n=5. Scale bar: 100 µm (left) and 50 μm (right). **C, D** Immunohistochemical staining and mean optical density (MOD) quantification of GABRD in spontaneous HCC tissues from Gabrd-KO vs. NTC (**C**) and Gabrd-OE vs. Control (**D**) , n=5. Scale bar: 100 µm (left) and 50 μm (right).

**Supplementary figure 4**

**Supplementary figure 4. GABRD drives HCC progression via EMT and JAK2-STAT3 activation**

**A.** Volcano plot showing differentially expressed genes (DEGs) in GABRD-OE HuH-7 cells compared with Control cells. **B.** Heatmap showing hierarchical clustering of the top significantly differentially expressed genes in GABRD-OE HuH-7 cells and control cells.

**Supplementary figure 5**

**Supplementary figure 5. GABRD drives HCC progression via EMT and JAK2-STAT3 activation**

**A, B.** Immunohistochemical staining and mean optical density (MOD) quantification of GABRD in pulmonary tissues of HCC lung metastasis from Gabrd-KO vs. NTC (**A**) and Gabrd-OE vs. Control (**B**) , n=5. Scale bar: 100 µm (left) and 50 μm (right). **C-F.** Immunohistochemical staining and mean optical density (MOD) quantification of E-cadherin (**C, D**) and Vimentin (**E, F**) in pulmonary tissues of HCC lung metastasis from Gabrd-KO vs. NTC (**C, E**) and Gabrd-OE vs. Control (**D, F**) , n=5. Scale bar: 100 µm (left) and 50 μm (right). **G.** Immunoblotting and quantification of phosphorylated JAK1, STAT1 and STAT5 in HCCLM3-GABRD-KO and HCCLM3-NTC cells, n=4. **H.** Immunoblotting and quantification of phosphorylated JAK1, STAT1 and STAT5 in HuH-7-GABRD and HuH-7-control cells, n=4.

**Supplementary figure 6.**

**Supplementary figure 6. JAK2-STAT3 inhibition reverses GABRD-mediated oncogenic effects in HCC**

**A.** Immunoblotting and quantification of p-JAK2, p-STAT3, T-JAK2, T-STAT3 and EMT markers including E-cadherin and Vimentin in Control-HuH7, GABRD-OE-HuH7, Control-HuH7 with WP1066 treatment and GABRD-OE-HuH7 cells with WP1066 treatment, n=4. **B.** Immunohistochemica staining and quantification of Ki-67 in xenograft tumors from GABRD-OE, GABRD-OE+NS and GABRD+WP, n=3, Scale bar: 100 µm (up) and 50 µm (below).

**Supplementary figure 7.**

**Supplementary figure 7. GABRD promotes HCC progression by binding to IL-10RA**

**A.** Immunoblotting and quantification of p-JAK2, p-STAT3, T-JAK2, T-STAT3 in in spontaneous HCC tissues from Control, GABRD-OE and GABRD-OE with IL-10RA neutralizing antibody treatment, n=4. **B.** Immunoblotting and qPCR analysis of GABRD protein and mRNA expression in HCCLM3-NTC , HCCLM3-GABRD-KO1 and HCCLM3-GABRD-KO2, n=4. **C.** Immunoblotting and qPCR analysis of IL-10RA protein and mRNA expression in HCCLM3-NTC , HCCLM3-IL-10RA-KO1 and HCCLM3-IL-10RA-KO2, n=4. **D.** Graphical abstract illustrating the oncogenic function of the GABRD/IL-10RA complex in HCC. The overexpression of GABRD promotes its interaction with the IL-10 receptor subunit IL-10RA at the cell membrane. This functional complex activates the downstream JAK2-STAT3 signaling pathway, leading to the induction of epithelial-mesenchymal transition and driving tumor progression and metastasis.
